# Synthesis, Structural Studies and Biological Evaluation of Connections of Thiosemicarbazide, 1,2,4-Triazole and 1,3,4-Thiadiazole with Palmitic Acid

**DOI:** 10.3390/molecules23040822

**Published:** 2018-04-03

**Authors:** Michał Jóźwiak, Karolina Stępień, Małgorzata Wrzosek, Wioletta Olejarz, Grażyna Kubiak-Tomaszewska, Anna Filipowska, Wojciech Filipowski, Marta Struga

**Affiliations:** 1 Department of Biochemistry and Pharmacogenomics, Faculty of Pharmacy, Medical University of Warsaw, 02-097 Warsaw, Poland; michal.jozwiak@wum.edu.pl (M.J.); malgorzata.wrzosek@wum.edu.pl (M.W.); wioletta.olejarz@wum.edu.pl (W.O.); grazyna.kubiak-tomaszewska@wum.edu.pl (G.K.-T.); 2 Department of Biochemistry, Second Faculty of Medicine, Medical University of Warsaw, 02-097 Warsaw, Poland; 3 Laboratory of Centre for Preclinical Research, Medical University of Warsaw, 02-097 Warsaw, Poland; karolina.stepien@wum.edu.pl; 4 Department of Pharmaceutical Microbiology, Medical University, 02-007 Warsaw, Poland; 5 Faculty of Automatic Control, Electronics and Computer Science, Silesian University of Technology, 44-100 Gliwice, Poland; wojciech.filipowski@polsl.pl; 6 Department of Biosensors and Processing of Biomedical Signals, Silesian University of Technology, 44-800 Zabrze, Poland; anna.filipowska@polsl.pl; 7 Department of Biochemistry, First Faculty of Medicine, Medical University of Warsaw, 02-097 Warsaw, Poland

**Keywords:** palmitic acid derivatives, thiosemicarbazides, 1,2,4-triazoles, 1,3,4-thiadiazoles, antimicrobial activity, quantitative structure-activity relationship

## Abstract

Thirty new derivatives of palmitic acid were efficiently synthesized. All obtained compounds can be divided into three groups of derivatives: Thiosemicarbazides (compounds **1**–**10**), 1,2,4-triazoles (compounds **1a**–**10a**) and 1,3,4-thiadiazoles (compounds **1b**–**10b**) moieties. ^1^H-NMR, ^13^C-NMR and MS methods were used to confirm the structure of derivatives. All obtained compounds were tested in vitro against a number of microorganisms, including Gram-positive cocci, Gram-negative rods and *Candida albicans*. Compounds **4**, **5**, **6**, **8** showed significant inhibition against *C. albicans*. The range of MIC values was 50–1.56 μg/mL. The halogen atom, especially at the 3rd position of the phenyl group was significantly important for antifungal activity. The biological activity against *Candida albicans* and selected molecular descriptors were used as a basis for QSAR models, that have been determined by means of multiple linear regression. The models have been validated by means of the Leave-One-Out Cross Validation. The obtained QSAR models were characterized by high determination coefficients and good prediction power.

## 1. Introduction

In recent years bacterial and fungal infections have become a clinical challenge due to issues such as transplantation, cancer therapy, HIV infections and the use of immunosuppressive agents [1,2]. In the treatment of infections azoles are widely used as antifungal agents. A triazole derivative, Fluconazole, was the first agent which had a high antifungal activity and showed low toxicity. Azoles inhibit the activity of lanosterol 14 α-demethylase enzyme (CYP51), which prevents the ergosterol synthesis in yeast and fungi. This depletion of ergosterol, and accumulation of lanosterol and other 14 α-methyl sterols causes the inhibition of growth of fungal cells [3,4]. The resistance of the most common strains of pathogenic fungi, *Candida albicans*, to fluconazole has been emerging rapidly [5,6]. To overcome this serious clinical problem and to enhance the activity of compounds, different derivatives of heterocyclic compounds are being synthesized containing not only 1,2,4-triazole arrangements, but also tetrazole [7], indole, benzimidazole, imidazole or 1,3,4-thiadiazole moieties [8]. To increase the antifungal activity of azoles, a piperazine ring can be introduced as side chains [9,10].

All mentioned heterocyclic moieties are common structures found in a large number of compounds which display biological activity. Chemical molecules containing triazole and thiadiazoles core play an important role in the medicinal chemistry and are known to be a very effective antibacterial and antifungal factors used to treat diseases caused by different microorganisms [11]. Compounds bearing heterocyclic moieties such as 1,2,4-triazole show, despite antifungal and antibacterial activity, anti-inflammatory properties [12] or can act as antioxidants [13]. Those containing 1,2,4-triazole and 1,3,4-thiadiazole had been also reported to exhibit significant anticancer properties [14,15,16].

To consider these chemical substances to be active compounds against different types of cells, the molecules must either interfere with plasma membranes or must enter the cells to affect metabolic reaction within it. Due to high membrane selectivity, numerous potentially active compounds cannot be transported across the membrane and therefore they do not display their properties.

Fatty acids and their derivatives have potential pharmacological activity [17,18,19,20]. The integrity of a fungal cell is provided by its plasma membrane, therefore insertion of fatty acids into bilayer can physically disrupt it and increase the fluidity [21]. Fatty acid derivatives containing heterocyclic moieties also tend to be biologically beneficial in combating microorganisms [22,23]. It is important to evaluate the antibacterial and antifungal effect of these five-membered heterocyclic compounds containing long alkyl chain as they show versatile pharmacological and biological activities. These compounds are expected to have advantages over the parent heterocyclic molecules; they may easily penetrate the cell membrane and they may disrupt the integrity of the cell [24].

Considering mentioned information on azoles and fatty acids properties, a series of palmitic acid derivatives was synthesized bearing 1,2,4-triazole and 1,3,4-thiadiazole moieties. The antibacterial and antifungal properties were evaluated and MIC values for all compounds were established.

## 2. Results and Discussion

### 2.1. Chemistry

**Scheme 1:**
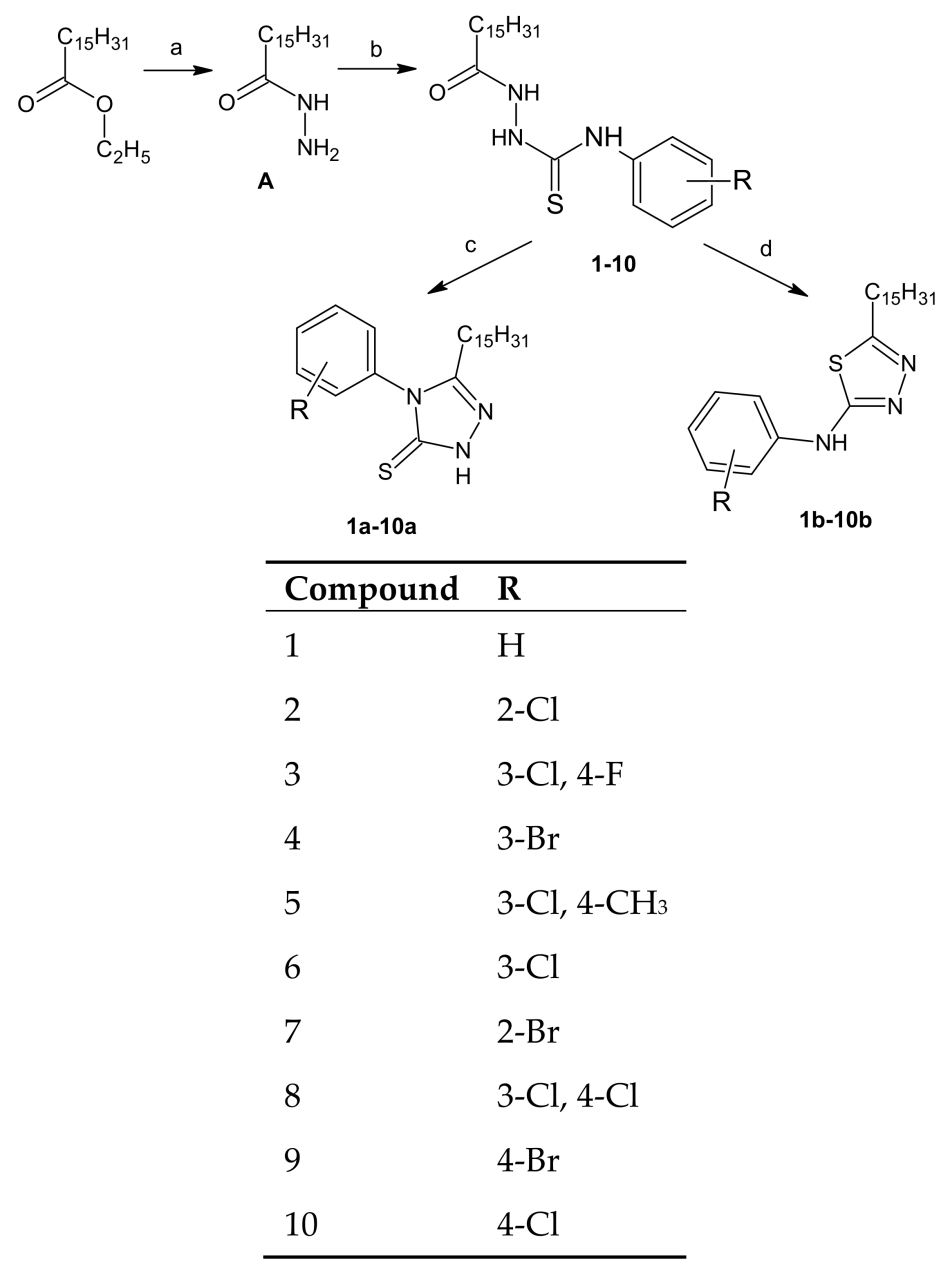
Synthesis route for derivatives of palmitic acid thiosemicarbazides. Reagents and conditions: (**a**) NH_2_NH_2_ 80%, EtOH, rt. 2 h (**b**) appropriate isothiocyanate, acetonitrile, reflux, 6 h (**c**) 2% NaOH, reflux (**d**) H_2_SO_4(conc.)_.

In this study, three series of compounds, series (**1**–**10**), which contain thiosemicarbazide, series (**1a**–**10a**), with 1,2,4-triazole moiety, and series (**1b**–**10b**), with 1,3,4-thiadiazole structure, were synthesized (Scheme 1).

In a first step, the ester (ethyl palmitate) was transformed into the palmitohydrazide (**A**) [25,26]. It was a starting material to obtain thiosemicarbazides. Derivatives of palmitic acid thiosemicarbazides were prepared in reaction of hydrazide (**A**) with an appropriate aryl isothiocyanate. In short, palmitohydrazide (**A**) and different isothiocyanates were allowed to react in boiling acetonitrile for 6 h. Obtained *N*-(phenylsubstituted)-2-palmitoyl hydrazinecarbothioamides (**1**–**10**) were a starting material to synthesize the 1,2,4-triazole (**1a**–**10a**) and 1,3,4-thiadiazole (**1b**–**10b**) palmitic acid derivatives. 1,2,4-Triazole (**1a**–**10a**) and 1,3,4-thiadiazole (**1b**–**10b**) were obtained using a method of cyclisation in basic (2% NaOH) and acidic (conc. H_2_SO_4_) conditions, respectively [27,28].

Spectral data (NMR, HRMS) of all compounds were in full agreement with the proposed structures (supplementary). The ^1^H-NMR spectrum for thiosemicarbazides (**1**–**10**) exhibited three broad singlets at δ 10.61–8.55 ppm. ^13^C-NMR revealed peaks at δ 169.41–169.69 ppm for C=S and peaks at δ 177.14–177.27 ppm for C=O.

^1^H-NMR spectra for triazole derivatives (**1a**–**10a**) are characterized by peaks for NH groups in the range δ 11.88–10.47 ppm, while ^13^C-NMR spectra for these derivatives show characteristic peaks in the range δ 168.34–168.42 ppm for groups C=S.

1,3,4-Thiadiazol-2-amine derivatives (**1b**–**10b**) have a NH group that is present in the ^1^H-NMR spectrum in the range δ 10.16–8.76 ppm.

### 2.2. Biological Activity

All obtained compounds were tested in vitro against 12 strains of bacteria, including Gram-positive cocci, Gram-negative rods and against two standard and two clinical isolates of *Candida albicans* using the serial microdilution assay. Microorganism used in this study were chosen on the basis of their common application in the antimicrobial tests for many substances, like antiseptic drugs, antibiotics or in the search of new antimicrobial agents [29,30]. All compounds were screened and MIC values for all derivatives were obtained.

#### 2.2.1. Antimicrobial Study

Results show, that all 30 tested compounds were inactive as antibacterial factors (MIC values above 100 μg/mL). Only two substances (**5b** and **8b**) from 1,3,4-thiadiazole series show moderate antibacterial activity against Gram-positive cocci. Derivative **5b** and **8b** MIC values were 50–12.5 μg/mL and 50–25 μg/mL, respectively. None of the compounds were active against Gram-negative bacteria.

#### 2.2.2. Antifungal Study

The antifungal activity of all obtained compounds was tested against standard strains and clinical isolates of *Candida albicans*.

Among three groups of tested compounds (thiosemicarbazides, 1,2,4-triazoles, 1,3,4-thiadiazoles) the thiosmicarbazide group (**1**–**10**) showed promising antifungal activity, but only four compounds were highly active against all tested *Candida* strains (compounds **4**, **5**, **6** and **8**). Compound **5** was found to be the most active with MIC value of 1.56 μg/mL for all strains, while the three remaining derivatives (**4**, **6** and **8**) showed weaker antifungal activity (MIC values of: 1.56 μg/mL against ATCC 10231, 50–25 μg/mL towards ATCC 30028, 12.5–3.125 μg/mL for clinical isolate 26 and 6.25–3.125 against clinical isolate 18).

*Candida* strain ATCC 10231 was the most sensitive against thiosemicarbazide derivatives (**1**–**10**). The MIC values were in the range of 12.5–1.56 μg/mL. The second strain, in terms of sensitivity, was the clinical isolate 18 (MIC range: 100–1.56 μg/mL).

All thiosemicarbazide derivatives contained a phenyl group in their structure. Compound **1** had unmodified phenyl ring, while compounds **2**–**10** were additionally substituted with at least one selected from the group consisting of chlorine, bromine and methyl group in the 2, 3 or 4 position.

Each of the four active thiosemicarbazides (**4**, **5**, **6**, **8**) had a halogen substituent in the 3-position in the phenyl ring. Derivative **4** containing bromine was the least active. Remaining three compounds had chlorine in position 3. Derivative **8** contained an additional chlorine substituent in position 4, while the most active compound **5** additionally had an electron donor substituent—methyl group in position 4.

Considering two other groups of compounds (1,2,4-triazoles, 1,3,4-thiadiazoles), only the 1,2,4-triazole derivative **5a**, obtained by cyclization of the most active thiosemicarbazide **5**, showed low level of antifungal activity (MIC 100–12.5 μg/mL).

All discussed results are presented in Table 1.

#### 2.2.3. Cytotoxic Studies

Cytotoxicity tests against three human cell lines were conducted. These were normal cells—human immortal keratinocyte cell line from adult human skin (HaCaT) and two tumor cell lines—human epithelial lung carcinoma cell line (A549) and human melanoma cell line (HTB-140). Two commonly used anticancer drugs—cisplatin and doxorubicin were used in this study as reference compounds. Assays were performed only for the three most active compounds (**5**, **6**, **8**) and for their corresponding derivatives **5a**, **6a**, **8a** and **5b**, **6b**, **8b** from each group of 1,2,4-triazoles and 1,3,4-thiadiazoles.

The cytotoxic activities of tested derivatives were specified by determining the IC_50_ (inhibitory concentrations), i.e., the concentrations of compounds, which inhibited cells’ viability and growth by 50% as compared to the control cells.

All tested derivatives were non-cytotoxic (IC_50_ > 100), which means that the antibacterial and antifungal activity was not related to cytotoxicity.

### 2.3. Quantitative Structure-Activity Relationships

For all tested derivatives of thiosemicarbazides, 1,2,4-triazoles and 1,3,4-thiadiazoles 19 molecular descriptors characterizing their physicochemical properties were determined.

Based on the calculated physicochemical parameters and microbiological assays, the similarity of tested compounds was analyzed and quantitative structure-activity relationships (QSAR) models describing the relationship between antifungal activity against *Candida albicans* ATCC 10231, *Candida albicans* ATCC 30028 and *Candida albicans* clinical isolate 26 and clinical isolate 18 strains were determined.

Quantitative structure-activity relationships (QSAR) modeling was performed using the multiple linear regression (MLR) regression method. The minimal inhibitory concentrations (MIC) obtained for tested fungal strains expressed as log (1/MIC (μM)) were dependent variables and molecular descriptors were independent variables. Obtained QSAR models were internally validated by Leave-One-Out Cross Validation (LOO) and Y-scrambling and externally validated by several metrics, including variance explained in external prediction Q^2^_(F1)_, Q^2^_(F2)_, Q^2^_(F3)_, and r^2^_m_ metrics based on the correlation of the observed and predicted response data with and without the intercept, and the criteria recommended by Golbraikh and Tropsha. The results of QSAR models are presented in Table 2.

4 QSAR models were determined for all tested strains, where antifungal activity depends on the following physicochemical parameters: logP—partition coefficient octanol/water, Rf—molar refractivity, µ—dipole moment, E_IA_—isolated atomic energy, E_E_—electronic energy, I_CC_—core-core interaction, HF—heat of formation.

For all the tested strains equations were obtained, where antimicrobial activity depends on molar refractivity (Rf) and on one descriptor characterizing electron properties from the group of descriptors described by the dependence E_T_ = E_B_ + E_IA_ = I_CC_ + E_E_. For each tested strain, simple linear equations can be obtained for each of above mentioned electron descriptors at the R > 0.6 level. Molar refractivity is, among others, considered as a measure of the mobility of electrons of a molecule or a measure of steric interactions of compound [31] and it defines the volume occupied by an atom or group of atoms. The presence of a strong correlation between Rf and biological activity of tested compounds may indicate, that the factor affecting this activity is the strength of binding of the compound to the polar surface [32,33].

Models, where one of the independent variables is logP, were obtained only for two of *C. albicans* strains: ATCC 10231 and clinical isolate 18. This parameter describes the lipophilicity of compounds and characterizes the ability of a compound to pass through biological membranes, i.e., the speed of distribution and absorption processes [31]. According to the Lipinski’s rule, the logP should not be greater than five (logP < 5) [34], however all compounds tested in this work show logP values above seven.

**Figure 1:**
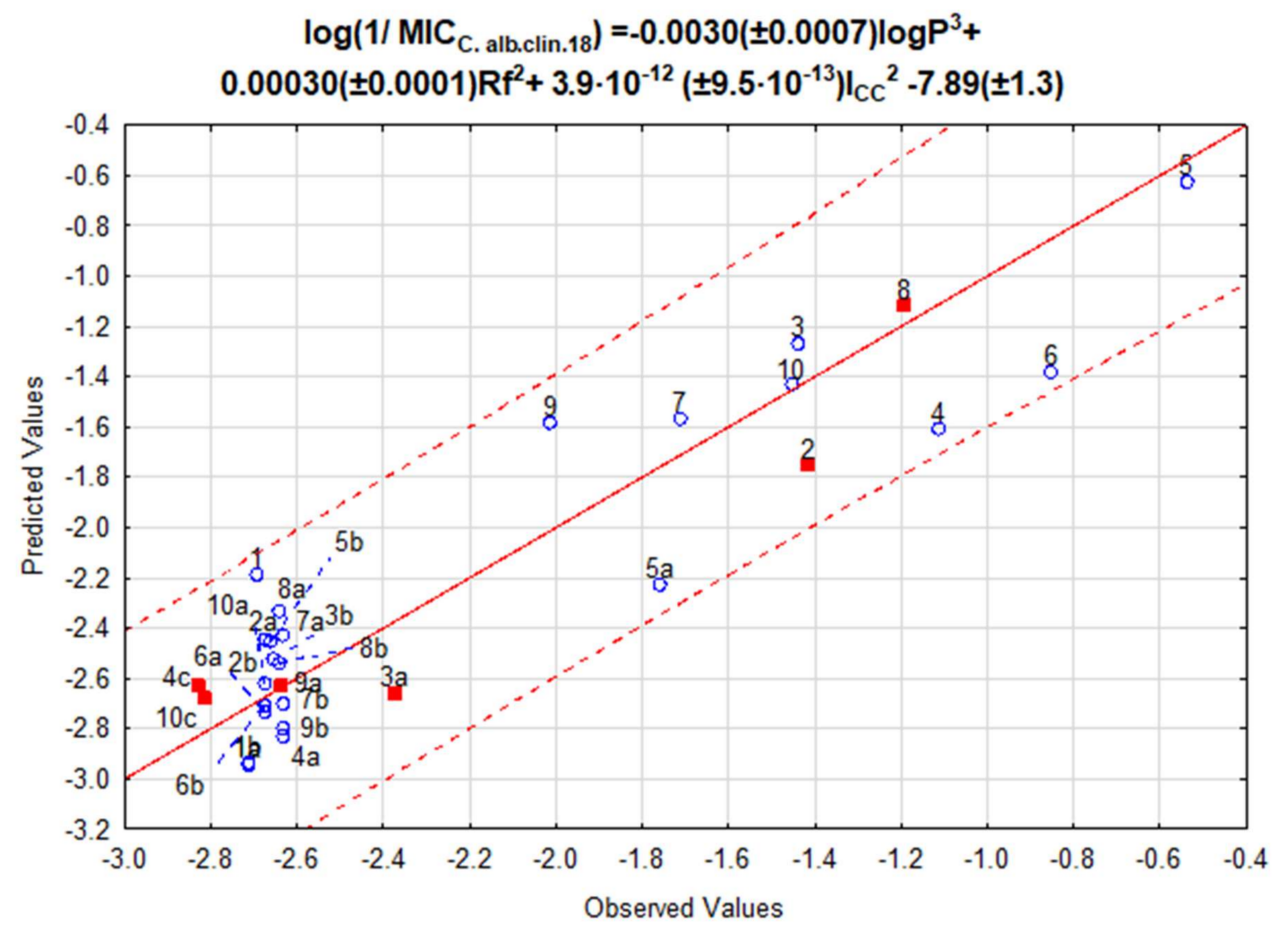
Correlation of activity against *Candida albicans* clinical isolate 18 and logP^3^, Rf^2^, I_CC_^2^. Dashed lines mark the area of predictability of the received models, which are described by the equations featured above the graphs. ▪—test set, ◦—training set.

**Figure 2:**
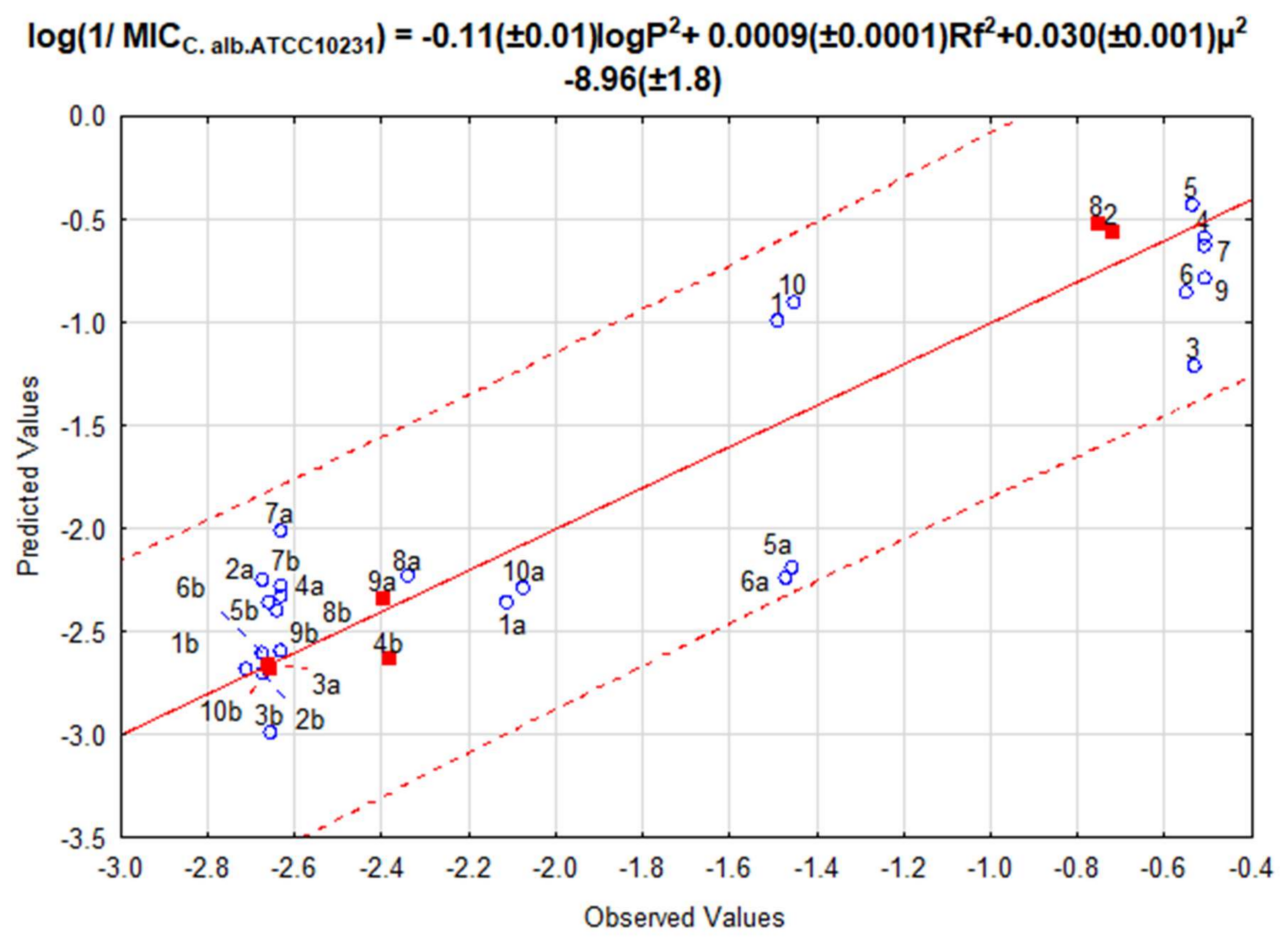
Correlation of activity against *Candida albicans* ATCC 10231 and logP^2^, RF^2^, µ^2^. Dashed lines mark the area of predictability of the received models, which are described by the equations featured above the graphs. ▪—test set, ◦—training set.

There is a positive linear correlation for the QSAR models obtained of such descriptors as Rf, HF, I_CC_^2^, E_E_, E_IA_, μ^2^ and negative linear correlation of logP^2^ descriptors against biological activity for selected fungal strains. All equations presented in Table 1 satisfy the acceptability and prediction conditions of the R^2^ > 0.6, Q^2^_LOO_ > 0.6, ${\mathrm{cR}}_{p}^{2}$ > 0.5, R^2^ − Q^2^_LOO_ < 0.3, Q^2^_(F1)_, Q^2^_(F2)_ and Q^2^_(F3)_ > 0.6, 0.85 ≤ k ≤ 1.15 or 0.85 ≤ k ≤ 1.15, (R^2^ − R_0_^2^)/R^2^ < 0.1 or (R^2^ − R’_0_^2^)/R^2^ < 0.1, $\bar{r_{m}{}^{2}}$ > 0.5 and ∆r^2^_m_ < 0.2 models [35,36,37,38]. The Figure 1 and Figure 2 show correlation between predicted and observed values for *C. albicans* strains characterized by the highest R^2^ determination coefficients.

**Figure 3:**
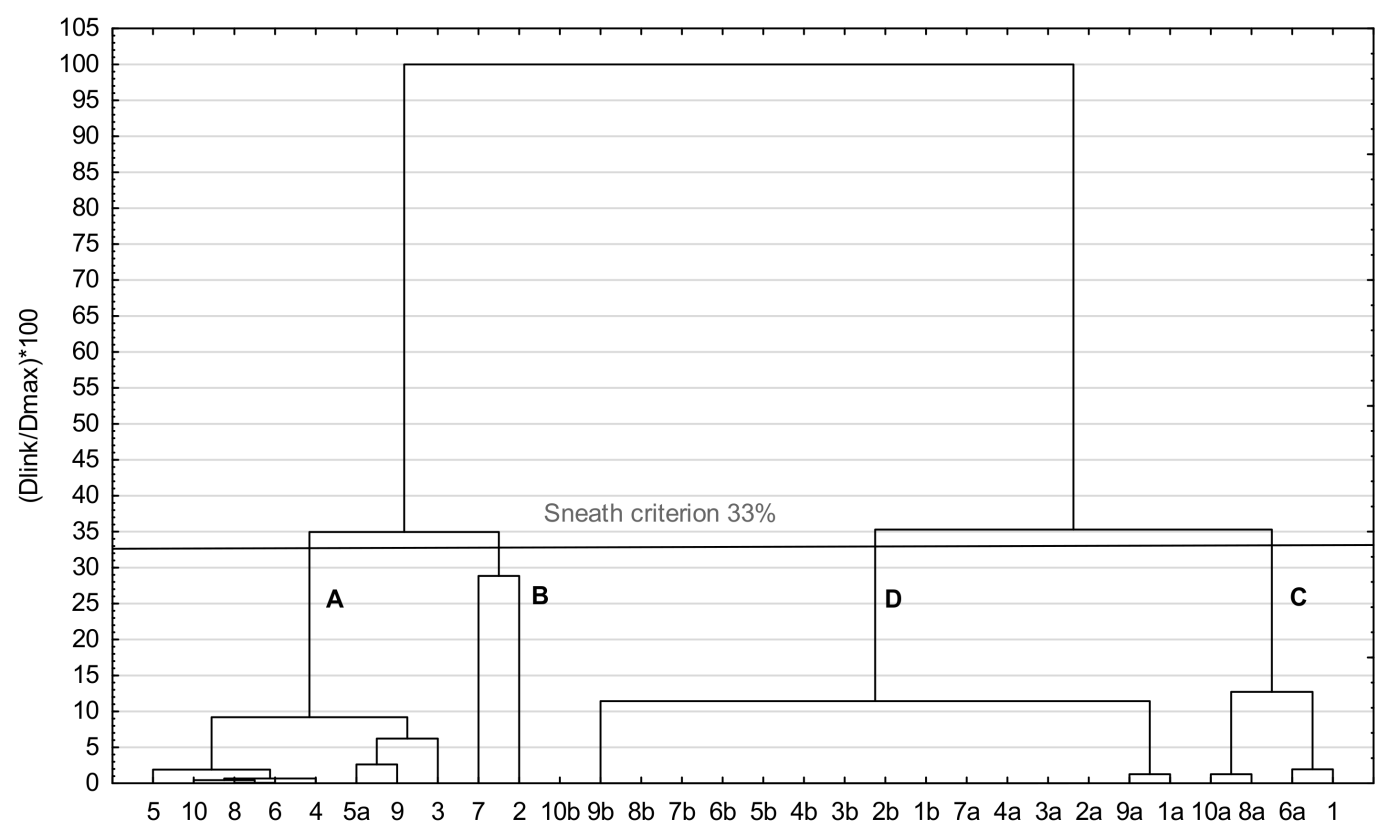
Dendrogram presenting the similarity of compounds in terms of their biological activity against the tested fungal strains: *Candida albicans* ATCC 10231, *Candida albicans* ATCC 3002, *Candida albicans* clinical isolate 26 and *Candida albicans* clinical isolate 18.

Dendrogram in Figure 3 presents the division of the tested compounds based on their biological activity for all 4 tested strains expressed as the minimum inhibitory concentration MIC (μg/mL). Based on 33% Sneath criterion compounds were divided into four groups (A–D). Group A consisted of compounds **5**, **10**, **8**, **6**, **4**, **15**, **9**, **3** showing strong activity for all tested strains (the average MIC value for all tested strains was lower than 62 μg/mL). In group B there were two compounds: **7** and **2** characterized by high biological activity, however not for all tested fungal strains (mean MIC value for all tested strains was in the range from 62.83 μg/mL to 81.64 μg/mL). Groups A and B contained almost all of the thiosemicarbazide derivatives tested and one triazole derivative (**5a**). In group C, there were compounds showing very low activity or having activity only against *Candida albicans* ATCC 10231 strain (average MIC value for all tested strains was in the range from 112.5 μg/mL to 128.1 μg/mL). Group D contained all compounds that do not show biological activity for the fungal strains tested.

## 3. Materials and Methods

### 3.1. Chemistry

Ethyl palmitate was supplied from Sigma-Aldrich. Isothiocyanates were purchased from Alfa Aesar or Sigma Aldrich. Acetonitrile, chloroform and methanol were supplied from POCh (Polskie Odczynniki Chemiczne, Gliwice, Poland). All chemicals were of analytical grade and were used without any further purification. Prior usage, dry acetonitrile was kept in crown cap bottles over anhydrous phosphorus pentoxide (Carl Roth, Karlsruhe, Germany). The NMR spectra were recorded on Varian VNMRS 300 Oxford NMR spectrometer (IET, Mundelein, IL, USA), operating at 300 MHz (^1^H-NMR, relax. delay 1000 s, pulse 30.0 degrees) and 75.4 MHz (^13^C-NMR, relax. delay 3700 s, pulse 45.0 degrees, proton-decoupled: Waltz-16 modulated). Chemical shifts (δ) were expressed in parts per million relative to tetramethylsilane used as the internal reference. Mass spectral ESI measurements were carried out on Waters ZQ Micro-mass instruments (Waters, Milford, MA, USA) with quadruple mass analyzer. The spectra were performed in the negative ion mode at a declustering potential of 40–60 V. The sample was previously separated on a UPLC column (C18) using UPLC ACQUITYTM system by Waters (Milford, MA, USA) connected with DPA detector. Flash chromatography was performed on Merck silica gel 60 (200–400 mesh) (Merck, Darmstadt, Germany) using chloroform eluent. Analytical TLC was carried out on silica gel F254 (Merck) plates (0.25 mm thickness).

#### 3.1.1. Palmitohydrazide (A)

Compound was synthesized and described previously [39,40].

#### 3.1.2. General Procedure for the Preparation of Palmitic Acid Thiosemicarbazide (**1**–**10**)

Palmitohydrazide (A 0.01 mol, 2.7 g) and 0.01 mol of appropriate isothiocyanate were refluxed in acetonitrile for 6 h. The product was filtered and dried. The solid residue was purified by column chromatography (chloroform:methanol 9.5:0.5 vol.).

*2-Palmitoyl-N-phenylhydrazinecarbothioamide* (**1**) Yield: 264 mg, 65%. Mp. 102–103 °C. ^1^H-NMR (DMSO-*d*_6_) δ (ppm): 9.81 (s, 1H, NH), 9.21 (s, 1H, NH), 8.72 (s, 1H, NH), 7.44 (d, *J* = 7.8 Hz, 2H, C_arom._), 7.37 (t, *J* = 7.65 Hz, 2H, C_arom._), 7.23 (t, *J* = 4.05 Hz, 1H, C_arom._), 2.35 (t, *J* = 7.5 Hz, 2H, CH_2_), 1.73–1.6 (m, 2H, CH_2_), 1.3–1.23 (m, 24H, CH_2_), 0.89 (t, *J* = 6.6 Hz, 3H, CH_3_). ^13^C-NMR (DMSO-d_6_) δ (ppm): 14.1, 22.7, 25.3, 29.1, 29.2, 29.35, 29.40, 29.57, 29.61, 29.66 (2C), 29.68, 31.9 (2C), 34.3, 124.2 (2C), 126.5, 129.3 (2C), 137.1, 169.7, 177.1. HRMS (ESI) calcd for C_23_H_39_N_3_OS [M − H]^−^: 404.2770; found 404.2769.

*N-(2-Chlorophenyl)-2-palmitoylhydrazinecarbothioamide* (**2**) Yield: 240 mg, 60%. Mp. 120–121 °C. ^1^H-NMR (DMSO-*d*_6_) δ (ppm): 10.61 (s, 1H, NH), 9.47 (s, 1H, NH), 8.86 (s, 1H, NH), 8.02 (d, *J* = 7.5 Hz, 1H, C_arom._), 7.44 (d, *J* = 7.8 Hz, 1H, C_arom._), 7.35–7.28 (m, 2H, C_arom._), 7.19 (t, *J* = 7.5 Hz, 1H, C_arom._), 2.37 (t, *J* = 7.2 Hz, 2H, CH_2_), 1.71–1.61 (m, 2H, CH_2_), 1.29–1.23 (m, 24H, CH_2_), 0.89 (t, *J* = 6.3 Hz, 3H, CH_3_). ^13^C-NMR (DMSO-d_6_) δ (ppm): 14.1, 22.7, 25.3, 29.1, 29.2, 29.35, 29.40, 29.57, 29.61, 29.66 (2C), 29.68, 31.9 (2C), 34.3, 124.2, 126.4, 129.4, 132.6 (2C) 137.1, 169.4, 177.2. HRMS (ESI) calcd for C_23_H_38_ClN_3_OS [M − H]^−^: 438.2346; found 438.2361.

*N-(3-Chloro-4-fluorophenyl)-2-palmitoylhydrazinecarbothioamide* (**3**) Yield: 284 mg, 62%. Mp. 119–120 °C. ^1^H-NMR (DMSO-*d*_6_) δ (ppm): 8.91 (s, 1H, NH), 8.77 (s, 1H, NH), 7.62 (t, *J* = 7.65 Hz, 1H, C_arom._), 7.42 (d, *J* = 8.1 Hz, 1H, C_arom._), 7.11 (t, J = 8.1 Hz, 1H, C_arom._), 5.26 (s, 1H, NH), 2.34 (t, *J* = 8.4 Hz, 2H, CH_2_), 1.76–1.60 (m, 2H, CH_2_), 1.32–1.23 (m, 24H, CH_2_), 0.87 (t, *J* = 6.45 Hz, 3H, CH_3_). ^13^C-NMR (DMSO-d_6_) δ (ppm): 14.1, 22.7, 25.8, 28.8, 28.9, 29.30, 29.31, 29.5, 29.58, 29.62 (2C), 29.64, 31.9 (2C), 34.3, 122.5, 124.7, 131.1, 134.5, 136.1, 148.4, 169.4, 177.2. HRMS (ESI) calcd for C_23_H_37_ClFN_3_OS [M − H]^−^: 456.2286; found 456.2297.

*N-(3-Bromophenyl)-2-palmitoylhydrazinecarbothioamide* (**4**) Yield: 310 mg, 64%. Mp. 113–114 °C. ^1^H-NMR (DMSO-*d*_6_) δ (ppm): 9.81 (s, 1H, NH), 9.05 (s, 1H, NH), 8.78 (s, 1H, NH), 7.68 (s,1H, C_arom._), 7.46 (d, *J* = 8.1 Hz, 1H, C_arom._), 7.34 (d, *J* = 8.1 Hz, 1H, C_arom._), 7.22 (t, *J* = 7.5 Hz, 1H, C_arom._), 2.34 (t, *J* = 7.65 Hz, 2H, CH_2_), 1.7–1.65 (m, 2H, CH_2_), 1.29–1.23 (m, 24H, CH_2_), 0.88 (t, *J* = 6.25 Hz, 3H, CH_3_). ^13^C-NMR (DMSO-d_6_) δ (ppm): 14.1, 22.7, 25.3, 29.1, 29.2, 29.35, 29.40, 29.57, 29.61, 29.66 (2C), 29.68, 31.9 (2C), 34.3, 123.4, 125.8 (2C), 127.6, 129.3, 137.1, 169.5, 177.2. HRMS (ESI) calcd for C_23_H_38_BrN_3_OS [M − H]^−^: 482.1875; found 482.1867.

*N-(3-Chloro-4-methylphenyl)-2-palmitoylhydrazinecarbothioamide* (**5**) Yield: 304 mg, 67%. Mp. 139–140 °C. ^1^H-NMR (DMSO-*d*_6_) δ (ppm): 9.90 (s, 1H, NH), 9.18 (s, 1H, NH), 8.77 (s, 1H, NH), 7.48 (s, 1H, C_arom._), 7.29 (d, *J* = 8.1 Hz, 1H, C_arom._), 7.19 (d, *J* = 8.1 Hz, 1H, C_arom._), 2.35 (t, *J* = 8.4 Hz, 2H, CH_2_), 2.34 (s, 3H, CH_3_), 1.76–1.60 (m, 2H, CH_2_), 1.32–1.23 (m, 24H, CH_2_), 0.87 (t, *J* = 6.45 Hz, 3H, CH_3_). ^13^C-NMR (DMSO-d_6_) δ (ppm): 14.1, 20.0, 22.7, 25.8, 28.8, 28.9, 29.29, 29.31, 29.5, 29.58, 29.62 (2C), 29.64, 31.9 (2C), 34.3, 122.5, 123.8, 124.7, 131.1, 134.5, 136.1, 169.4, 177.2. HRMS (ESI) calcd for C_24_H_40_ClN_3_OS [M − H]^−^: 452.2237; found 452.2528.

*N-(3-Chlorophenyl)-2-palmitoylhydrazinecarbothioamide* (**6**) Yield: 246 mg, 56%. Mp. 98–99 °C. ^1^H-NMR (DMSO-*d*_6_) δ (ppm): 10.19 (s, 1H, NH), 9.28 (s, 1H, NH), 9.04 (s, 1H, NH), 7.56 (s, 1H, C_arom._), 7.41 (d, *J* = 7.8 Hz, 1H, C_arom._), 7.31 (d, *J* = 6.6 Hz, 1H, C_arom._), 7.18 (t, *J* = 7.8 Hz, 1H, C_arom._), 2.36 (t, *J* = 7.5 Hz, 2H, CH_2_), 1.7–1.55 (m, 2H, CH_2_), 1.29–1.22 (m, 24H, CH_2_), 0.87 (t, *J* = 6.15 Hz, 3H, CH_3_). ^13^C-NMR (DMSO-d_6_) δ (ppm): 14.1, 22.7, 25.3, 29.1, 29.2, 29.35, 29.40, 29.57, 29.61, 29.66 (2C), 29.68, 31.9 (2C), 34.3, 124.6, 126.5, 128.3, 129.3, 136.1, 137.1, 169.5, 177.3. HRMS (ESI) calcd for C_23_H_38_ClN_3_OS [M − H]^−^: 438.2346; found 438.2361.

*N-(2-Bromophenyl)-2-palmitoylhydrazinecarbothioamide* (**7**) Yield: 334 mg, 69%. Mp. 105–106 °C. ^1^H-NMR (DMSO-*d*_6_) δ (ppm): 10.0 (s, 1H, NH), 9.15 (s, 1H, NH), 8.58 (s, 1H, NH), 7.92 (d, *J* = 7.5 Hz, 1H, C_arom._), 7.6 (d, *J* = 8.1 Hz, 1H, C_arom._), 7.36 (t, *J* = 7.8 Hz, 1H, C_arom._), 7.12 (t, *J* = 7.8 Hz, 1H, C_arom._), 2.35 (t, *J* = 7.35 Hz, 2H, CH_2_), 1.7–1.6 (m, 2H, CH_2_), 1.3–1.21 (m, 24H, CH_2_), 0.88 (t, *J* = 6.6 Hz, 3H, CH_3_). ^13^C-NMR (DMSO-*d*_6_) δ (ppm): 14.1, 22.7, 25.2, 29.1, 29.2, 29.35, 29.40, 29.57, 29.61, 29.66 (2C), 29.68, 31.9 (2C), 34.3, 122.4, 128.1, 130.5, 132.2, 132.9, 137.1, 169.7, 177.1. HRMS (ESI) calcd for C_23_H_38_BrN_3_OS [M − H]^−^: 482.1875; found 482.1867.

*N-(3,4-Dichlorophenyl)-2-palmitoylhydrazinecarbothioamide* (**8**) Yield: 342 mg, 72%. Mp. 103–104 °C. ^1^H-NMR (DMSO-*d*_6_) δ (ppm): 9.31 (s, 1H, NH), 9.09 (s, 1H, NH), 8.94 (s, 1H, NH), 7.71 (d, *J* = 2.4 Hz, 1H, C_arom._), 7.43 (d, *J* = 2.1 Hz, 1H, C_arom._), 7.38 (s, 1H, C_arom._), 2.35 (t, *J* = 9 Hz, 2H, CH_2_), 1.73–1.62 (m, 2H, CH_2_), 1.32–1.22 (m, 24H, CH_2_), 0.88 (t, *J* = 6.75 Hz, 3H, CH_3_).^13^C-NMR (DMSO-*d*_6_) δ (ppm): 14.1, 22.7, 25.3, 29.1, 29.2, 29.35, 29.40, 29.57, 29.61, 29.66 (2C), 29.68, 31.9 (2C), 34.3, 121.1, 129.3, 129.4, 131.3, 135.4, 137.2, 169.6, 177.2. HRMS (ESI) calcd for C_23_H_37_Cl_2_N_3_OS [M − H]^−^: 472.1991; found 472.1985.

*N-(4-Bromophenyl)-2-palmitoylhydrazinecarbothioamide* (**9**) Yield: 339 mg, 70%. Mp. 120–121 °C. ^1^H-NMR (DMSO-*d*_6_) δ (ppm): 9.8 (s, 1H, NH), 9.09 (s, 1H, NH), 8.82 (s, 1H, NH), 7.47 (d, *J* = 6.9 Hz, 2H, C_arom._), 7.38 (d, *J* = 6.9 Hz, 2H, C_arom._), 2.34 (t, *J* = 7.65 Hz, 2H, CH_2_), 1.7–1.6 (m, 2H, CH_2_), 1.32–1.21 (m, 24H, CH_2_), 0.88 (t, *J* = 6.75 Hz, 3H, CH_3_).^13^C-NMR (DMSO-*d*_6_) δ (ppm): 14.1, 22.7, 25.3, 29.1, 29.2, 29.35, 29.40, 29.57, 29.61, 29.66 (2C), 29.68, 31.9 (2C), 34.3, 127.7, 131.7 (2C), 132.1 (2C), 137.4, 169.5, 177.1. HRMS (ESI) calcd for C_23_H_38_BrN_3_OS [M − H]^−^: 482.1875; found 482.1867.

*N-(4-Chlorophenyl)-2-palmitoylhydrazinecarbothioamide* (**10**) Yield: 268 mg, 61%. Mp. 115–116 °C. ^1^H-NMR (DMSO-*d*_6_) δ (ppm): 10.05 (s, 1H, NH), 9.15 (s, 1H, NH), 8.55 (s, 1H, NH), 7.43 (d, *J* = 7.1 Hz, 2H, C_arom._), 7.32 (d, *J* = 7.1 Hz, 2H, C_arom._), 2.34 (t, *J* = 7.5 Hz, 2H, CH_2_), 1.68–1.58 (m, 2H, CH_2_), 1.32–1.19 (m, 24H, CH_2_), 0.88 (t, *J* = 6.75 Hz, 3H, CH_3_).^13^C-NMR (DMSO-*d*_6_) δ (ppm): 14.1, 22.7, 25.2, 29.1, 29.2, 29.35, 29.41, 29.57, 29.61, 29.66 (2C), 29.68, 31.9 (2C), 34.3, 129.1 (2C), 131.1 (2C), 133.7, 137.1, 169.6, 177.2. HRMS (ESI) calcd for C_23_H_38_ClN_3_OS [M − H]^−^: 438.2346; found 438.2361.

#### 3.1.3. General Procedure for Derivatives of 3-Pentadecyl-1*H*-1,2,4-Triazole-5(4*H*)-Thione (**1a**–**10a**)

*N*-(Substituted-phenyl)-2-palmitoyl-hydrazinecarbothioamide (0.01 mol) was refluxed with 2% NaOH solution (40–50 mL) for 4 h. After cooling the solution was neutralized with dilute hydrochloric acid. The precipitated compound was filtered and then crystalized from ethanol.

*3-Pentadecyl-4-phenyl-1H-1,2,4-triazole-5(4H)-thione* (**1a**) Yield: 279 mg, 72%. Mp. 89–90 °C. ^1^H-NMR (DMSO-*d*_6_) δ (ppm): 11.79 (s, 1H, NH), 7.59–7.53 (m, 3H, C_arom._), 7.34 (d, *J* = 2.1 Hz, 1H, C_arom._), 7.31 (d, *J* = 1.5 Hz, 1H, C_arom._), 2.46 (t, *J* = 7.65 Hz, 2H, CH_2_), 1.59–1.5 (m, 2H, CH_2_), 1.25–1.19 (m, 24H, CH_2_), 0.87 (t, *J* = 6.6 Hz, 3H, CH_3_). ^13^C-NMR (DMSO-*d*_6_) δ (ppm): 14.1, 22.7, 25.8, 26.0, 28.8, 29.0, 29.3, 29.3, 29.5, 29.6 (2C), 29.6 (2C), 31.9 (2C), 127.9 (2C), 129.8 (2C), 130.0, 133.5, 153.0, 168.3. HRMS (ESI) calcd for C_23_H_37_N_3_S [M − H]^−^: 386.2630; found 386.2629.

*4-(2-Chlorophenyl)-3-pentadecyl-1H-1,2,4-triazole-5(4H)-thione* (**2a**) Yield: 338 mg, 80%. Mp. 139–140 °C. ^1^H-NMR (DMSO-*d*_6_) δ (ppm): 10.47 (s, 1H, NH), 7.64 (d, *J* = 7.8 Hz, 1H, C_arom._), 7.56–7.46 (m, 2H, C_arom._), 7.39 (d, *J* = 7.2 Hz, 1H, C_arom._), 2.48–2.31 (m, 2H, CH_2_), 1.68–1.53 (m, 2H, CH_2_), 1.26–1.21 (m, 24H, CH_2_), 0.87 (t, *J* = 6.75 Hz, 3H, CH_3_).^13^C-NMR (DMSO-*d*_6_) δ (ppm): 14.0, 22.7, 25.8, 26.0, 28.8, 29.0, 29.27, 29.32, 29.5, 29.61 (2C), 29.63 (2C), 31.9 (2C), 124.3, 126.4, 129.4, 132,6 (2C) 137.1, 153.1, 168.4. HRMS (ESI) calcd for C_23_H_36_ClN_3_S [M − H]^−^: 420.2240; found 420.2223.

*4-(3-Chloro-4-fluorophenyl)-3-pentadecyl-1H-1,2,4-triazole-5(4H)-thione* (**3a**) Yield: 321 mg, 73%. Mp. 90–91 °C. ^1^H-NMR (DMSO-*d*_6_) δ (ppm): 11.88 (s, 1H, NH), 7.43 (dd, *J* = 2.2 Hz, *J* = 2.2 Hz, 1H, C_arom._), 7.34 (t, *J* = 7.65 Hz, 1H, C_arom._), 7.27−7.22 (m, 1H, C_arom._), 2.46 (t, *J* = 7.65 Hz, 2H, CH_2_), 1.63–1.53 (m, 2H, CH_2_), 1.26–1.20 (m, 24H, CH_2_), 0.87 (t, *J* = 6.75 Hz, 3H, CH_3_). ^13^C-NMR (DMSO-*d*_6_) δ (ppm): 14.1, 22.7, 25.8, 25.9, 28.8, 29.0, 29.29, 29.32, 29.5, 29.58 (2C), 29.62 (2C), 31.9 (2C), 126.2, 128.4, 131.9, 135.6, 138.6, 148.8, 152.9, 168.4. HRMS (ESI) calcd for C_23_H_35_ClFN_3_S [M − H]^−^: 438.2146; found 438.2157.

*4-(3-Bromophenyl)-3-pentadecyl-1H-1,2,4-triazole-5(4H)-thione* (**4a**) Yield: 317 mg, 68%. Mp. 101–102 °C. ^1^H-NMR (DMSO-*d*_6_) δ (ppm): 11.80 (s, 1H, NH), 7.43 (dd, *J* = 1.5 Hz, *J* = 1.5 Hz, 1H, C_arom._), 7.50 (t, *J* = 1.8 Hz, 1H, C_arom._), 7.45 (t, *J* = 8.1 Hz, 1H, C_arom._), 7.30 (dd, *J* = 1.5 Hz, *J* = 1.5 Hz, 1H, C_arom._), 2.46 (t, *J* = 7.65 Hz, 2H, CH_2_), 1.63–1.53 (m, 2H, CH_2_), 1.26–1.20 (m, 24H, CH_2_), 0.87 (t, *J* = 6.75 Hz, 3H, CH_3_). ^13^C-NMR (DMSO-*d*_6_) δ (ppm): 14.0, 22.7, 25.8, 26.0, 28.8, 29.0, 29.27, 29.32, 29.5, 29.61 (2C), 29.63 (2C), 31.9 (2C), 123.4, 125.8 (2C), 127.6, 129.3, 137.1, 153.2, 168.4. HRMS (ESI) calcd for C_23_H_36_BrN_3_S [M − H]^−^: 464.1735; found 464.1727.

*4-(3-Chloro-4-methylphenyl)-3-pentadecyl-1H-1,2,4-triazole-5(4H)-thione* (**5a**) Yield: 270 mg, 62%. Mp. 94–95 °C. ^1^H-NMR (DMSO-*d*_6_) δ (ppm): 11.85 (s, 1H, NH), 7.43 (dd, *J* = 0.3 Hz, *J* = 0.3 Hz, 1H, C_arom._), 7.33 (d, *J* = 2.1 Hz, 1H, C_arom._), 7.14 (dd, *J* = 2.4 Hz, *J* = 2.4 Hz,1H, C_arom._), 2.46 (s, 3H, CH_3_), 2.46 (t, *J* = 7.65 Hz, 2H, CH_2_), 1.62–1.52 (m, 2H, CH_2_), 1.26–1.20 (m, 24H, CH_2_), 0.87 (t, *J* = 6.75 Hz, 3H, CH_3_). ^13^C-NMR (DMSO-*d*_6_) δ (ppm): 14.1, 20.0, 22.7, 25.8, 25.9, 28.8, 29.0, 29.29, 29.32, 29.5, 29.58 (2C), 29.62 (2C), 31.9 (2C), 126.2, 128.4, 131.9, 135.6 (2C), 138.6, 152.9, 168.4. HRMS (ESI) calcd for C_24_H_38_ClN_3_S [M − H]^−^: 434.2397; found 434.2388.

*4-(3-Chlorophenyl)-3-pentadecyl-1H-1,2,4-triazole-5(4H)-thione* (**6a**) Yield: 312 mg, 74%. Mp. 97–98 °C. ^1^H-NMR (DMSO-*d*_6_) δ (ppm): 11.79 (s, 1H, NH), 7.53–7.51 (m, 1H, C_arom._), 7.35 (s, 1H, C_arom._), 7.28–7.24 (m, 2H, C_arom._), 2.46 (t, *J* = 7.65 Hz, 2H, CH_2_), 1.62–1.52 (m, 2H, CH_2_), 1.24–1.21 (m, 24H, CH_2_), 0.87 (t, *J* = 6.6 Hz, 3H, CH_3_). ^13^C-NMR (DMSO-*d*_6_) δ (ppm): 14.1, 22.7, 25.8, 25.9, 28.8, 29.0, 29.29, 29.32, 29.5, 29.58 (2C), 29.62 (2C), 31.9 (2C), 124.6, 126.5, 128.4, 129.4, 136.2, 137.2, 153.0, 168.4. HRMS (ESI) calcd for C_23_H_36_ClN_3_S [M − H]^−^: 420.2240; found 420.2223.

*4-(2-Bromophenyl)-3-pentadecyl-1H-1,2,4-triazole-5(4H)-thione* (**7a**) Yield: 327 mg, 70%. Mp. 128–129 °C. ^1^H-NMR (DMSO-*d*_6_) δ (ppm): 11.63 (s, 1H, NH), 7.8 (d, *J* = 7.8 Hz, 1H, C_arom._), 7.56–7.36 (m, 3H, C_arom._), 2.51–2.27 (m, 2H, CH_2_), 1.64–1.55 (m, 2H, CH_2_), 1.23–1.21 (m, 24H, CH_2_), 0.87 (t, *J* = 6.6 Hz, 3H, CH_3_). ^13^C-NMR (DMSO-*d*_6_) δ (ppm): 14.1, 22.7, 25.8, 25.9, 28.8, 29.0, 29.29, 29.32, 29.5, 29.58 (2C), 29.62 (2C), 31.9 (2C), 122.5, 128.2, 130.6, 132.2, 133.0, 137.1, 153.1, 168.4. HRMS (ESI) calcd for C_23_H_36_BrN_3_S [M − H]^−^: 464.1735; found 464.1727.

*4-(3,4-Dichlorophenyl)-3-pentadecyl-1H-1,2,4-triazole-5(4H)-thione* (**8a**) Yield: 338 mg, 74%. Mp. 84–85 °C. ^1^H-NMR (DMSO-*d*_6_) δ (ppm): 11.7 (s, 1H, NH), 7.66 (d, *J* = 8.4 Hz, 2H, C_arom._), 7.46 (s, 1H, C_arom._), 7.23 (d, *J* = 8.4 Hz, 1H, C_arom._), 2.46 (t, *J* = 7.5 Hz, 2H, CH_2_), 1.6–1.53 (m, 2H, CH_2_), 1.23–1.21 (m, 24H, CH_2_), 0.87 (t, *J* = 6 Hz, 3H, CH_3_). ^13^C-NMR (DMSO-*d*_6_) δ (ppm): 14.1, 22.7, 25.8, 25.9, 28.8, 29.0, 29.29, 29.32, 29.5, 29.58 (2C), 29.62 (2C), 31.9 (2C), 122.5, 128.2, 130.6, 132.2, 13.0, 137.1, 153.1, 168.4. HRMS (ESI) calcd for C_23_H_35_Cl_2_N_3_S [M − H]^−^: 454.1851; found 454.1845.

*4-(4-Bromophenyl)-3-pentadecyl-1H-1,2,4-triazole-5(4H)-thione* (**9a**) Yield: 294 mg, 63%. Mp. 79–80 °C. ^1^H-NMR (DMSO-*d*_6_) δ (ppm): 11.68 (s, 1H, NH), 7.7 (d, *J* = 8.4 Hz, 2H, C_arom._), 7.21 (d, *J* = 8.4 Hz, 2H, C_arom._), 2.46 (t, *J* = 7.65 Hz, 2H, CH_2_), 1.68–1.51 (m, 2H, CH_2_), 1.23–1.21 (m, 24H, CH_2_), 0.87 (t, *J* = 6.6 Hz, 3H, CH_3_). ^13^C-NMR (DMSO-*d*_6_) δ (ppm): 14.1, 22.7, 25.8, 25.9, 28.8, 29.0, 29.29, 29.32, 29.5, 29.58 (2C), 29.62 (2C), 31.9 (2C), 127.7, 131.8 (2C), 132.2 (2C), 137.5, 153.0, 168.4. HRMS (ESI) calcd for C_23_H_36_BrN_3_S [M − H]^−^: 464.1735; found 464.1727.

*4-(4-Chlorophenyl)-3-pentadecyl-1H-1,2,4-triazole-5(4H)-thione* (**10a**) Yield: 291 mg, 69%. Mp. 66–67 °C. ^1^H-NMR (DMSO-*d*_6_) δ (ppm): 11.62 (s, 1H, NH), 7.24 (d, *J* = 8.4 Hz, 2H, C_arom._), 6.57 (d, *J* = 8.4 Hz, 2H, C_arom._), 2.46 (t, *J* = 7.65 Hz, 2H, CH_2_), 1.68–1.51 (m, 2H, CH_2_), 1.23–1.21 (m, 24H, CH_2_), 0.88 (t, *J* = 6.6 Hz, 3H, CH_3_). ^13^C-NMR (DMSO-*d*_6_) δ (ppm): 14.1, 22.7, 25.8, 25.9, 28.8, 29.0, 29.2, 29.3, 29.5, 29.58 (2C), 29.62 (2C), 31.9 (2C), 129.2 (2C), 131.2 (2C), 133.8, 137.1, 153.0, 168.4. HRMS (ESI) calcd for C_23_H_36_ClN_3_S [M − H]^−^: 420.2240; found 420.2223.

#### 3.1.4. General Procedure for Derivatives of 5-Pentadecyl-*N*-(Substituted Phenyl)-1,3,4-Thiadiazol-2-Amine (**1b**–**10b**)

*N*-(Substituted-phenyl)-2-palmitoyl-hydrazinecarbothioamide (0.01 mol) was mixed for 4 h with concentrated sulfuric acid (0.5 mL). Then crushed ice was added to the solution. After cooling the solution neutralized with dilute NaOH. The precipitated compound was filtered and dried. The solid residue was purified by column chromatography (chloroform:methanol 9.5:0.5 vol.).

*5-Pentadecyl-N-phenyl-1,3,4-thiadiazol-2-amine* (**1b**) Yield: 186 mg, 48%. Mp. 97–98 °C. ^1^H-NMR (DMSO-*d*_6_) δ (ppm): 9.52 (s, 1H, NH), 7.59–7.53 (m, 3H, C_arom._), 7.40 (d, *J* = 2.1 Hz, 1H, C_arom._), 7.38 (d, *J* = 1.5 Hz, 1H, C_arom._), 2.46 (t, *J* = 7.65 Hz, 2H, CH_2_), 1.68–1.63 (m, 2H, CH_2_), 1.25–1.19 (m, 24H, CH_2_), 0.87 (t, *J* = 6.6 Hz, 3H, CH_3_). ^13^C-NMR (DMSO-*d*_6_) δ (ppm): 14.1, 22.7, 25.7, 26.0, 28.5, 28.8, 29.1, 29.3, 29.5, 29.61 (2C), 29.63 (2C), 31.9 (2C), 127.4, 130.2 (2C), 135.4 (2C), 140.8, 158.1, 173.0. HRMS (ESI) calcd for C_23_H_37_N_3_S [M − H]^−^: 386.2630; found 386.2629.

*N-(2-Chlorophenyl)-5-pentadecyl-1,3,4-thiadiazol-2-amine* (**2b**) Yield: 224 mg, 53%. Mp. 66–67 °C. ^1^H-NMR (DMSO-*d*_6_) δ (ppm): 9.59 (s, 1H, NH), 7.63 (dd, *J* = 2.7 Hz, *J* = 2.7 Hz, 1H, C_arom._), 7.30–7.25 (m, 1H, C_arom._), 7.10 (t, *J* = 8.7 Hz, 1H, C_arom._), 2.89 (t, *J* = 7.65 Hz, 2H, CH_2_), 1.71–1.61 (m, 2H, CH_2_), 1.25–1.19 (m, 24H, CH_2_), 0.87 (t, *J* = 6.6 Hz, 3H, CH_3_). ^13^C-NMR (DMSO-*d*_6_) δ (ppm): 14.1, 22.7, 25.8, 26.0, 28.8, 29.0, 29.27, 29.32, 29.5, 29.61 (2C), 29.63 (2C), 31.9 (2C), 124.3, 126.5, 129.9, 133.0 (2C) 140.8, 158.1, 173.1. HRMS (ESI) calcd for C_23_H_36_ClN_3_S [M − H]^−^: 420.2240; found 420.2223.

*N-(3-Chloro-4-fluorophenyl)-5-pentadecyl-1,3,4-thiadiazol-2-amine* (**3b**) Yield: 246 mg, 56%. Mp. 84–85 °C. ^1^H-NMR (DMSO-d_6_) δ (ppm): 8.75 (s, 1H, NH), 7.50 (dd, *J* = 1.8 Hz, *J* = 1.8 Hz, 1H, C_arom._), 7.39–7.28 (m, 2H, C_arom._), 7.38 (d, *J* = 1.5 Hz, 1H, C_arom._), 2.87 (t, *J* = 7.65 Hz, 2H, CH_2_), 1.76–1.66 (m, 2H, CH_2_), 1.25–1.19 (m, 24H, CH_2_), 0.87 (t, *J* = 6.6 Hz, 3H, CH_3_).^13^C-NMR (DMSO-d_6_) δ (ppm): 14.1, 22.7, 25.8, 25.9, 28.5, 28.8, 29.1, 29.3, 29.57, 29.62 (2C), 29.7 (2C), 30.4, 31.9, 128.0, 132.3, 137.7, 138. 9, 139.1, 148.8, 158.5, 173.2. HRMS (ESI) calcd for C_23_H_35_ClFN_3_S [M − H]^−^: 438.2146; found 438.2157.

*N-(3-Bromophenyl)-5-pentadecyl-1,3,4-thiadiazol-2-amine* (**4b**) Yield: 229 mg, 49%. Mp. 82–83 °C. ^1^H-NMR (DMSO-*d*_6_) δ (ppm): 8.75 (s, 1H, NH), 7.87 (d, *J* = 2.2 Hz1H, C_arom._), 7.67–7.55 (m, 2H, C_arom._), 7.38 (d, *J* = 2.4 Hz, 1H, C_arom._), 2.85 (t, *J* = 7.65 Hz, 2H, CH_2_), 1.76–1.66 (m, 2H, CH_2_), 1.25–1.19 (m, 24H, CH_2_), 0.87 (t, *J* = 6.6 Hz, 3H, CH_3_). ^13^C-NMR (DMSO-*d*_6_) δ (ppm): 14.1, 22.6, 25.8, 26.0, 28.8, 29.0, 29.2, 29.3, 29.5, 29.62 (2C), 29.63 (2C), 31.9 (2C), 123.4, 125.8 (2C), 127.7, 129.4, 140.8, 158.5, 173.2. HRMS (ESI) calcd for C_23_H_36_BrN_3_S [M − H]^−^: 464.1735; found 464.1727.

*N-(3-Chloro-4-methylphenyl)-5-pentadecyl-1,3,4-thiadiazol-2-amine* (**5b**) Yield: 262 mg, 60%. Mp. 116–117 °C. ^1^H-NMR (DMSO-*d*_6_) δ (ppm): 8.76 (s, 1H, NH), 7.43 (dd, *J* = 0.3 Hz, *J* = 0.3 Hz, 1H, C_arom._), 7.38 (d, *J* = 2.1 Hz, 1H, C_arom._), 7.14 (dd, *J* = 2.4 Hz, *J* = 2.4 Hz,1H, C_arom._), 2.79 (t, *J* = 7.65 Hz, 2H, CH_2_), 2.45 (s, 3H, CH_3_), 1.62–1.52 (m, 2H, CH_2_), 1.26–1.20 (m, 24H, CH_2_), 0.87 (t, *J* = 6.75 Hz, 3H, CH_3_). ^13^C-NMR (DMSO-*d*_6_) δ (ppm): 14.1, 19.9, 22.7, 25.8, 25.9, 28.5, 28.8, 29.1, 29.3, 29.57, 29.62 (2C), 29.7 (2C), 30.4, 31.9, 128.0, 132.3, 133.6, 137.8, 138. 9, 139.1, 158.5, 173.2. HRMS (ESI) calcd for C_24_H_38_ClN_3_S [M − H]^−^: 434.2397; found 434.2388.

*N-(3-Chlorophenyl)-5-pentadecyl-1,3,4-thiadiazol-2-amine* (**6b**) Yield: 241 mg, 57%. Mp. 94–95 °C. ^1^H-NMR (DMSO-*d*_6_) δ (ppm): 10.16 (s, 1H, NH), 7.45–7.44 (m, 1H, C_arom._), 7.29–7.27 (m, 1H, C_arom._), 7.05 – 7.01 (m, 1H, C_arom._), 2.99 (t, *J* = 7.65 Hz, 2H, CH_2_), 1.83–1.73 (m, 2H, CH_2_), 1.29–1.21 (m, 24H, CH_2_), 0.87 (t, *J* = 6.6 Hz, 3H, CH_3_).^13^C-NMR (DMSO-*d*_6_) δ (ppm): 14.1, 22.7, 25.8, 26.0, 28.8, 29.0, 29.30, 29.34, 29.5, 29.59 (2C), 29.63 (2C), 31.9 (2C), 125.1, 127.5, 129.4, 130.4, 137.2, 139.2, 158.8, 173.2. HRMS (ESI) calcd for C_23_H_36_ClN_3_S [M − H]^−^: 420.2240; found 420.2223.

*N-(2-Bromophenyl)-5-pentadecyl-1,3,4-thiadiazol-2-amine* (**7b**) Yield: 238 mg, 51%. Mp. 66–67 °C. ^1^H-NMR (DMSO-*d*_6_) δ (ppm): 8.76 (s, 1H, NH), 7.9 (d, *J* = 8.1 Hz, 1H, C_arom._), 7.6 (d, *J* = 7.8 Hz, 1H, C_arom._) 7.34 (t, *J* = 7.8 Hz, 1H, C_arom._), 7.00 (t, *J* = 7.65 Hz, 1H, C_arom._), 2.95 (t, *J* = 7.65 Hz, 2H, CH_2_), 1.76–1.69 (m, 2H, CH_2_), 1.29–1.21 (m, 24H, CH_2_), 0.87 (t, *J* = 6.6 Hz, 3H, CH_3_). ^13^C-NMR (DMSO-*d*_6_) δ (ppm): 14.1, 22.7, 25.8, 26.0, 28.8, 29.0, 29.31, 29.34, 29.5, 29.62 (2C), 29.65 (2C), 31.9 (2C), 123.5, 129.1, 131.6, 133.2, 135.0, 140.1, 158.5, 173.2. HRMS (ESI) calcd for C_23_H_36_BrN_3_S [M − H]^−^: 464.1735; found 464.1727.

*N-(3,4-Dichlorophenyl)-5-pentadecyl-1,3,4-thiadiazol-2-amine* (**8b**) Yield: 242 mg, 53%. Mp. 64–65 °C. ^1^H-NMR (DMSO-*d*_6_) δ (ppm): 9.08 (s, 1H, NH), 7.60 (d, *J* = 8.4 Hz, 2H, C_arom._), 7.48 (s, 1H, C_arom._), 7.33 (d, *J* = 6.9 Hz, 1H, C_arom._), 2.92 (t, *J* = 7.5 Hz, 2H, CH_2_), 1.69–1.67 (m, 2H, CH_2_), 1.23–1.21 (m, 24H, CH_2_), 0.87 (t, *J* = 6 Hz, 3H, CH_3_). ^13^C-NMR (DMSO-*d*_6_) δ (ppm): 14.1, 22.7, 25.8, 26.0, 28.8, 29.0, 29.3, 29.4, 29.5, 29.59 (2C), 29.64 (2C), 31.7 (2C), 123.5, 129.2, 131.5, 133.2, 134.0, 139.1, 158.6, 173.2. HRMS (ESI) calcd for C_23_H_35_Cl_2_N_3_S [M − H]^−^: 454.1851; found 454.1845.

*N-(4-Bromophenyl)-5-pentadecyl-1,3,4-thiadiazol-2-amine* (**9b**) Yield: 229 mg, 49%. Mp. 67–68 °C. ^1^H-NMR (DMSO-*d*_6_) δ (ppm): 8.76 (s, 1H, NH), 7.68 (d, *J* = 8.4 Hz, 2H, C_arom._), 6.79 (d, *J* = 8.4 Hz, 2H, C_arom._), 2.87 (t, *J* = 7.65 Hz, 2H, CH_2_), 1.67–1.51 (m, 2H, CH_2_), 1.25–1.21 (m, 24H, CH_2_), 0.87 (t, *J* = 6.6 Hz, 3H, CH_3_). ^13^C-NMR (DMSO-*d*_6_) δ (ppm): 14.1, 22.7, 25.8, 26.0, 28.8, 29.0, 29.30, 29.32, 29.5, 29.61 (2C), 29.63 (2C), 31.9 (2C), 128.7, 132.8 (2C), 133.2 (2C), 140.1, 158.0, 173.1. HRMS (ESI) calcd for C_23_H_36_BrN_3_S [M − H]^−^: 464.1735; found 464.1727.

*N-(4-Chlorophenyl)-5-pentadecyl-1,3,4-thiadiazol-2-amine* (**10b**) Yield: 249 mg, 59%. Mp. 66–67 °C. ^1^H-NMR (DMSO-*d*_6_) δ (ppm): 8.86 (s, 1H, NH), 7.34 (d, *J* = 8.4 Hz, 2H, C_arom._), 6.67 (d, *J* = 8.4 Hz, 2H, C_arom._), 2.86 (t, *J* = 7.65 Hz, 2H, CH_2_), 1.69–1.51 (m, 2H, CH_2_), 1.25–1.21 (m, 24H, CH_2_), 0.87 (t, *J* = 6.6 Hz, 3H, CH_3_). ^13^C-NMR (DMSO-*d*_6_) δ (ppm): 14.1, 22.7, 25.8, 26.0, 28.8, 29.0, 29.31, 29.34, 29.5, 29.59 (2C), 29.64 (2C), 31.9 (2C), 123.2 (2C), 132.2 (2C), 134.8, 140.1, 158.46, 173.0. HRMS (ESI) calcd for C_23_H_36_ClN_3_S [M − H]^−^: 420.2240; found 420.2223.

### 3.2. Biological Assay

#### 3.2.1. In Vitro Evaluation of Antimicrobial Activity

Microorganisms used in this study were as follows: Gram-positive bacteria: *Staphylococcus aureus* NCTC 4163, *S. aureus* ATCC 25923, *S. aureus* ATCC 6538, *S. aureus* ATCC 29213, *S. epidermidis* ATCC 12228, *S. epidermidis* ATCC 35984, *Bacillus subtilis* ATCC 6633, *Enterococcus hirae* ATCC 9341, *Enterococcus faecalis* ATCC 29212; Gram-negative rods: *E. coli* ATCC 8196, *Pseudomonas aeruginosa* ATCC 15442, *Bordetella bronchiseptica* ATCC 4617 and yeasts: *Candida albicans* ATCC 10231, *C. albicans* ATCC 30028, *C. albicans* clinical isolate 26, *C. albicans* clinical isolate 18.

All microorganisms used were obtained from the collection of the Department of Pharmaceutical Microbiology, Medical University of Warsaw, Poland.

#### 3.2.2. Media, Growth Conditions and Antimicrobial Assay

Minimal Inhibitory Concentration (MIC) was tested by the twofold serial microdilution method (in 96-well microtiter plates) using Mueller-Hinton Broth medium (Beckton Dickinson, Franklin Lakes, NJ, USA) for bacteria or RPMI-1640 medium for *Candida* species according to CLSI guidelines [41,42]. The stock solution of tested compounds was prepared in DMSO and diluted in sterile water. Concentrations of tested agents ranged from 1.56 to 200 μg/mL for bacteria strains and from 1.56 to 100 μg/mL for fungi. The final inoculum of all studied bacteria was 10^5^ CFU/mL (colony forming units per mL) and 0.5–2.5 × 10^5^ CFU/mL for fungi. Minimal inhibitory concentrations (the lowest concentration of the tested agent that prevents visible growth of a microorganism) were read after 18 h (bacteria) or 24 h (yeasts) of incubation at 35 °C.

#### 3.2.3. Cytotoxicity Studies

Human immortal keratinocyte cell line from adult human skin (HaCaT). Human epithelial lung carcinoma cell line (A549) and Human melanoma cell line (HTB-140) were bought from American Type Culture Collection (Rockville, USA) and cultured in Dulbecco’s Modified Eagle’s Medium (DMEM) supplemented with 1% antibiotics (penicillin and streptomycin), and 10% heat-inactivated FBS-fetal bovine serum (Gibco Life Technologies, Waltham, MA, USA), at 37 °C and 5% CO_2_ atmosphere. Cells were passaged using trypsin-EDTA (Gibco Life Technologies, Waltham, MA, USA) and cultured in 96-well plates (1 × 10^4^ cells per well). Experiments were conducted in DMEM with 2% FBS.

#### 3.2.4. MTT Cytotoxicity Assay

The cell viability was assessed by determination of MTT salt 3-(4,5-dimethylthiazol-2-yl)-2,5- diphenyltetrazolium bromide, converted by mitochondrial dehydrogenase. The cells were incubated for 72 h in 96-well plates with given concentration of tested compound, and subsequently for another 4 h with 0.5 mg/mL of MTT solution, which in live cells—under the effect of mitochondrial dehydrogenase—converts to insoluble formazan. The converted dye was then solubilized with the use of 0.04 M HCl in absolute isopropanol. The absorbance of solubilized formazan was measured spectrophotometrically at 570 nm (using Epoch microplate reader, BioTek Inc., Winooski, VT, USA), equipped with Gen5 software (BioTech Instruments, Inc., Biokom, Winooski, VT, USA). Cell viability was presented as a percent of MTT reduction in the treated cells versus the control (cells incubated in serum-free DMEM without studied compounds). The relative MTT level (%) was calculated as (A)/(B) × 100, where (A) is the absorbance of the tested sample and (B) means the absorbance of the control sample containing untreated cells. Decreasing of the relative MTT level indicates decreased cell viability.

### 3.3. QSAR Modeling

#### 3.3.1. Physicochemical Parameters

Conformational search and physicochemical parameters were calculated using HyperChem ver. 7.5 [43]. Optimized Potentials for Liquid Simulations (OPLS) force field were used for extensive conformational search performed at molecular mechanics level. The most stable structures obtained were subsequently optimized to the closest local minimum at the semiempirical level using PM3 parametrizations. Convergence criteria were set to 0.1 and 0.01 kcal·mol^−1^·Å^−1^ for OPLS and PM3 calculations, respectively [44,45]. The selected descriptors were then used to develop a QSAR models.

#### 3.3.2. QSAR Models

QSAR models have been determined by means of multiple linear regression (MLR) [46]. Comparison of multiple linear regression equations (Y = B_0_ + B_1_·X_1_ + B_2_·X_2_ .... + B_N_·X_N_) [47] have been performed with the Statistica 12.0 software [48]. Equation coefficients were calculated by means of the classic least squares method [49], in which the sum of squares of differences between the theoretical value and deviations of empirical value for variable Y and is the lowest. The statistical calculations were conducted at confidence level of 95% (*p* < 0.05). The analyses were performed by means of multiple backward regression which entailed successively rejecting the least statistically significant (having the highest *p* value) structural parameters (molecular descriptors). Statistical verification of the model was performed by means of *T*-test and Fisher–Snedecor *F*-test. *T*-test is used to verify significance of each parameter of a model (independent, explanatory variables), and F-test is used for checking statistical significance of the entire model [50]. The following multiple linear regression assumptions were verified for statistically significant equations:

- Model linearity,
- coincidence condition,
- uniform dispersion (homoscedasticity) condition,
- normal distribution of model residue.

The coincidence condition entails checking if the sign of the coefficient of correlation between the explanatory variable X_j_ and the response variable Y is the same as the sign of the B_j_ coefficient in the multiple linear regression model (with multiple explanatory variables) of the X_j_ variable.

The homoscedasticity condition has been analyzed based on diagrams of residual dispersion in relation to observed variables and based on the Lagrange Multiplier Test.

Normal distribution of the dependent variable Y and model residuals were verified by means of the Shapiro–Wilk test [51]. Every independent variable used in a QSAR model is related to at least five compounds [47].

#### 3.3.3. Model Validation

The model has been internally validated by means of the Leave-One-Out Cross Validation (LOO CV). Successively one compound, utilized to validate the resulting model, was eliminated from the data set used as a basis for model (*n* − 1). The Q^2^_LOO_ validation coefficient was calculated from the formula [52,53]:

(1) $$Q^{2}=1-\frac{\sum_{i=1}^{n}{\left(y_{exp,i}-y_{pred,i}\right)}^{2}}{\sum_{i=1}^{n}{\left(y_{exp,i}-\;y_{ave,i}\right)}^{2}}$$

where: *y_exp,i_*—experimental output value for the i-th compound, *y_pred,i_*—predicted output value for the i-th compound, *y_ave,i_*—average value for the output variable without the *i*-th compound.

For all models, validation in the form of a Y-randomization test (Scrambling model) was also performed to check, whether the selection of descriptors used to create QSAR models was not random. The test is based on the multiple replacements of the biological activity values used in MLR with random values. The deviation in the values of the squared mean correlation coefficient of the randomized model (Rr^2^) from the squared correlation coefficient of the nonrandom model (R^2^) is reflected in the value of cR_p_^2^ parameter computed from the following equation [54]:

(2) $${\mathrm{cR}}_{p}^{2}=\;R\sqrt{R^{2}-R_{r}^{2}}$$

The threshold value of cR_p_^2^ is 0.5. For a QSAR model having the corresponding value above the stated limit, it might be considered that the model is not obtained by chance [37,38].

External validation of the models was also conducted. The parameters of predictive models such as Q^2^_(F1)_ [38], Q^2^_(F2)_ and Q^2^_(F3)_ [55] were calculated.

The parameters k, k’, R^2^_0_, R’^2^_0_ for determining the external predictability of QSAR models were calculated using methods described in Golbraikh and Tropsha [56].

Internally validation of the prediction model was also carried out by calculating the $\bar{r_{m}{}^{2}}$, Δr_m_^2^ parameters described by Roy and et al. [57]. The obtained QSAR models should meet the assumptions $\bar{r_{m}^{2}}$ > 0.5 and Δr_m_^2^ < 0.2 [57].

#### 3.3.4. Cluster Analysis

Cluster analysis is used to identify a relatively similar, homogeneous group of objects (compounds) in the measured features space. In this paper, the agglomerative hierarchical cluster analysis complete linkage method (squared Euclidean distance) was conducted to find a group of compounds characterized by similar biological activity or similar physicochemical parameters. Data standardization was the first stage of cluster analysis. Its purpose was to increase the influence of data with minor deviation and decrease the influence of data with major deviation. Furthermore, the standardization procedure eliminates the influence of different units of measurement and renders the data dimensionless [58].

## 4. Conclusions

We successfully synthesized three groups of compounds: Thiosemicarbazides (compounds **1**–**10**), 1,2,4-triazoles (compounds **1a**–**10a**) and 1,3,4-thiadiazoles (compounds **1b**–**10b**) to determine their microbiological activity and cytotoxicity. Obtained compounds did not show significant activity against Gram-positive cocci and Gram-negative bacteria.

Thiosemicarbazide derivatives exhibited potent to moderate activity against *Candida albicans*. Compound **5** presented the strongest potency against all tested strains of *C. albicans*, with MIC values 1.56 μg/mL. Derivatives **4**, **6** and **8** showed diverse activity for different strains of fungi. The MIC value for *C. albicans* ATCC 10231 was 1.56 μg/mL, for *C. albicans* 30028 was in the range 50–25 μg/mL, for *C. albicans* isolate 26 it was 2.5–3.125 μg/mL and for *C. albicans* isolate 18—6.25–3.125 μg/mL.

Biological activity was closely related to the presence of a halogen substituent at the 3-position of the aromatic ring; substituents in other positions did not affect the antimicrobial activity. Chloride atoms turn out to have the strongest influence.

The statistical analysis allowed to determine 4 QSAR models, where the antifungal activity depends on the following physicochemical parameters: E_IA_, E_E_, I_CC_, μ, logP, Rf, HF. The obtained models can be used to predict the biological activity against the tested fungal strains for the new thiosemicarbazide, triazole and thiadiazole derivatives, as the effect of E_IA_, E_E_, I_CC_ descriptors on the antifungal activity against all tested strains was indicated.

## Figures and Tables

**Table 1:** Antifungal activity against *Candida albicans* stains (MIC values in μg/mL).

Compound	MIC/μg/mL			
	*Candida albicans* ATCC 10231	*Candida albicans* ATCC 30028	*Candida albicans* Clinical Isolate 26	*Candida albicans* Clinical Isolate 18
**1**	12.5	100	>100	>100
**2**	1.56	100	>100	25
**3**	1.56	100	50	12.5
**4**	1.56	50	12.5	6.25
**5**	1.56	1.56	1.56	1.56
**6**	1.56	25	3.125	3.125
**7**	1.56	>100	25	25
**8**	1.56	25	12.5	6.25
**9**	1.56	50	50	50
**10**	12.5	25	25	12.5
**1a**	50	>100	>100	>100
**2a**	>100	>100	>100	>100
**3a**	>100	>100	>100	>100
**4a**	>100	>100	>100	>100
**5a**	12.5	25	100	25
**6a**	12.5	50	>100	>100
**7a**	>100	>100	>100	>100
**8a**	100	100	100	>100
**9a**	100	>100	>100	>100
**10a**	50	100	100	>100
**1b**	>100	>100	>100	>100
**2b**	>100	>100	>100	>100
**3b**	>100	>100	>100	>100
**4b**	>100	>100	>100	>100
**5b**	>100	>100	>100	>100
**6b**	>100	>100	>100	>100
**7b**	>100	>100	>100	>100
**8b**	>100	>100	>100	>100
**9b**	>100	>100	>100	>100
**10b**	>100	>100	>100	>100
Ref. *	0.25–0.125	0.25–0.125	0.25–0.125	0.25–0.125

* Ref.—Fluconazole.

**Table 2:** Formulas describing the relationship of activity against *Candida albicans* ATCC 10231, *Candida albicans* clinical isolate 18 and molecular descriptors (training set = 80% of whole dataset, test set = 20% of whole dataset).

C. albicans Strain	QSAR Equation	Modeling n_tr_ = 24	Internal Validation	External Validation n_test_ = 6	Golbraikh&Tropsha
Candida albicans ATCC 10231	log(1/MIC_C. alb.ATCC10231_) = 0.0001(±0.00005)E_IA_ + 0.19 (±0.05)Rf − 0.039(±0.006)HF − 11.84(±3.9)	R = 0.889	Q^2^_LOO_ = 0.733 R^2^_Yscr_ = 0.146 Q^2^_Yscr_ = −0.097 ${\mathrm{cR}}_{p}^{2}$ = 0.713	Q^2^_(F1)_ = 0.890	k = 1.013 k’ = 0.964 R^2^_0_ = 0.891 R’^2^_0_ = 0.838
		R^2^ = 0.790		Q^2^_(F2)_ = 0.890	
		R^2^_adj._= 0.758		Q^2^_(F3)_ = 0.867	
		F = 25.1		RMSE_ext_ = 0.321	
		*p* = 5.6·10^-7^		$\bar{r_{m}^{2}}$ = 0.717	
		RMSE_tr_ = 0.419		Δr^2^_m_ = 0.111	
	log(1/MIC_C. alb.ATCC10231_) = −0.11(±0.01)logP^2^ + 0.00090(±0.0001)Rf^2^ + 0.030(±0.01)µ^2^ − 8.96(±1.8)	R = 0.901	Q^2^_LOO_ = 0.750 R^2^_Yscr_ = 0.089 Q^2^_Yscr_ = −0.167 ${\mathrm{cR}}_{p}^{2}$ = 0.766	Q^2^_(F1)_ = 0.879	k = 1.005 k’ = 0.989 R^2^_0_ = 0.845 R’^2^_0_ = 0.778
		R^2^ = 0.812		Q^2^_(F2)_ = 0.879	
		R^2^_adj._= 0.784		Q^2^_(F3)_ = 0.904	
		F = 28.8		RMSE_ext_ = 0.208	
		*p* = 1.9·10^−7^		$\bar{r_{m}^{2}}$ = 0.687	
		RMSE_tr_ = 0.419		Δr^2^_m_ = 0.145	
Candida albicans clinical isolate 18	log(1/MIC_C. alb.clin.18_) = −0.003(±0.0007)logP^3^ + 0.0003(±0.0001)Rf^2^ + 3.9·10^−^^12^(±1·10^−^^12^)I_CC_^2^ − 7.89 (±1.1)	R = 0.918	Q^2^_LOO_ = 0.774 R^2^_Yscr_ = 0.122 Q^2^_Yscr_ = −0.115 ${\mathrm{cR}}_{p}^{2}$ = 0.814	Q^2^_(F1)_ = 0.879	k = 1.005 k’ = 0.989 R^2^_0_ = 0.880 R’^2^_0_ = 0.902
		R^2^ = 0.843		Q^2^_(F2)_ = 0.879	
		R^2^_adj._= 0.819		Q^2^_(F3)_ = 0.904	
		F = 35.7		RMSE_ext_ = 0.208	
		*p* = 3.2·10^−8^		$\bar{r_{m}^{2}}$ = 0.806	
		RMSE_tr_ = 0.292		Δr^2^_m_ = 0.095	
	log(1/MIC_C. alb.clin.18_) = 4.9·10^−12^(±7·10^-13^)E_E_^2^ − 6.94(±0.7)	R = 0.831	Q^2^_LOO_ = 0.673 R^2^_Yscr_ = 0.057 Q^2^_Yscr_ = −0.041 ${\mathrm{cR}}_{p}^{2}$ = 0.630	Q^2^_(F1)_ = 0.640	k = 1.009 k’ = 0.967 R^2^_0_ = 0.641 R’^2^_0_ = 0.605
		R^2^ = 0.691		Q^2^_(F2)_ = 0.641	
		R^2^_adj._= 0.677		Q^2^_(F3)_ = 0.712	
		F = 49.2		RMSE_ext_ = 0.360	
		*p* = 4.87·10^−7^		$\bar{r_{m}^{2}}$ = 0.539	
		RMSE_tr_ = 0.390		Δr^2^_m_ = 0.064	

*n*_tr_—the number of samples in training set, R—multilinear correlation coefficient, R^2^—determination coefficient, R^2^_adj_—adjusted determination coefficient, RMSE_tr_—root mean square error in fitting, F—F-value; R^2^_Yscr_—average R^2^ of 25 random models, Q^2^_Yscr_—average R^2^ of 25 random models, ${\mathrm{cR}}_{p}^{2}=\sqrt{R^{2}-R_{\mathrm{Yscr}}{}^{2}}$, Q^2^_LOO_—determination coefficient of LOO validation, *n*_test_—the number of samples in test set, RMSE_ext_—root mean square error in test set, Q^2^_(F1),_ Q^2^_(F2)_ and Q^2^_(F3)_—variance explained in test set, concordance correlation coefficient; $\bar{r_{m}{}^{2}}$ and ∆r^2^_m_—average and delta r^2^_m_ values, respectively (criteria described by Roy), k and k′—slopes of the regression line over external data, R^2^_0_ and R’^2^_0_—R^2^ values in Golbraikh and Tropsha criteria.

## Supplementary Materials

The following are available online, Figures S1–S30: ^1^H-NMR spectra of the products **1**–**10**, **1a**–**10a**, **1b**–**10b**. Figures S31–S36: ^13^C-NMR spectra of the selected products **1**–**10**, **1a**–**10a**, **1b**–**10b**.
