# Supplementary material for: Synthesis, Structural Studies and Biological Evaluation of Connections of Thiosemicarbazide, 1,2,4-Triazole and 1,3,4-Thiadiazole with Palmitic Acid
Source: Molecules. 2018 Apr 3;23(4):822. doi: 10.3390/molecules23040822 (PMC6017783; doi:10.3390/molecules23040822)
Supplement: Supplementary file 1 [file molecules-23-00822-s001.pdf]

ELECTRONIC SUPPLEMENTARY MATERIAL

Synthesis, Structural Studies and Biological Evaluation of Connections of Thiosemicarbazide, 1,2,4-Triazole and 1,3,4-Thiadiazole with Palmitic Acid.

Michał Jóźwiak[1,2,5], Karolina Stępień[3,5], Małgorzata Wrzosek[1,5], Wioletta Olejarz[1,5], Grażyna Kubiak-Tomaszewska[1], Anna Filipowska[6], Wojciech Filipowski[7], and Marta Struga[4,5,*].

[1] Department of Biochemistry and Pharmacogenomics, Faculty of Pharmacy, Medical University of Warsaw, 02-097 Warsaw, Poland; malgorzata.wrzosek@wum.edu.pl (M.W.)
[2] Department of Biochemistry, Second Faculty of Medicine, Medical University of Warsaw, 02-097 Warsaw, Poland; michal.jozwiak@wum.edu.pl (M.J.)
[3] Department of Pharmaceutical Microbiology, Medical University, 02-007 Warsaw, Poland; karolina.stepien@wum.edu.pl (K.S.)
[4] Department of Biochemistry, First Faculty of Medicine, Medical University of Warsaw, 02-097 Warsaw, Poland; marta.struga@wum.edu.pl (M.S.)
[5] Laboratory of Centre for Preclinical Research, Medical University of Warsaw, 02-097 Warsaw, Poland; marta.struga@wum.edu.pl (M.S.)
[6] Department of Biosensors and Processing of Biomedical Signals, Silesian University of Technology, 44-800 Zabrze, Poland; anna.filipowska@polsl.pl (A.F.)
[7] Faculty of Automatic Control, Electronics and Computer Science, Silesian University of Technology, 44-100 Gliwice, Poland; wojciech.filipowski@polsl.pl (W.F.)

* Correspondence: marta.struga@wum.edu.pl

CONTENTS

^{1}H NMR spectra of the products 1-10, 1a-10a, 1b-10b 2-16

^{13}C NMR spectra of the selected products 1-10, 1a-10a, 1b-10b 17-19

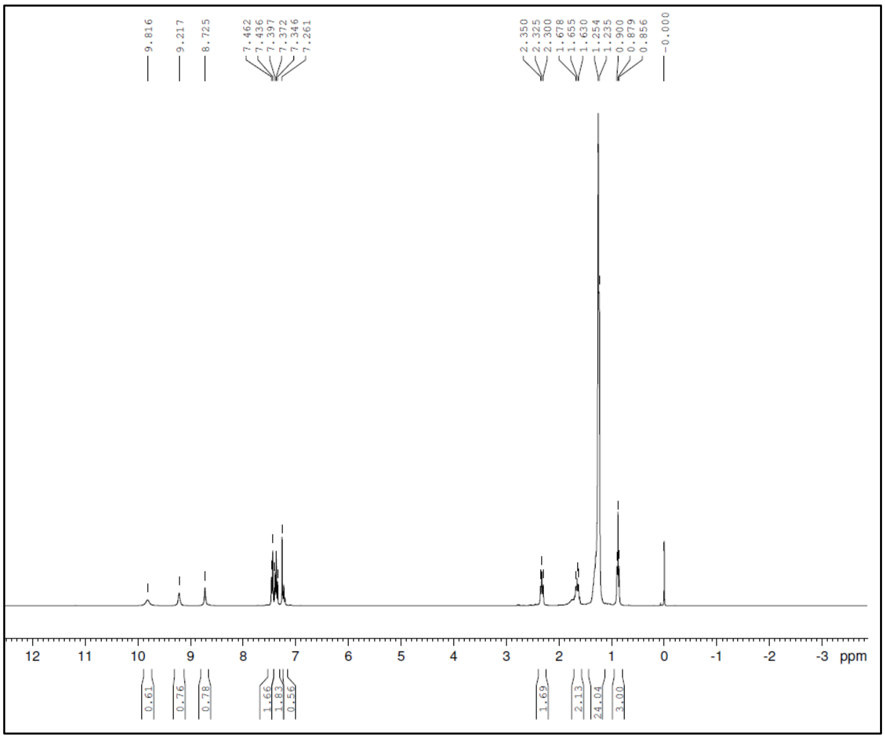

Figure S1. ^{1}H NMR spectrum of the product **1.**

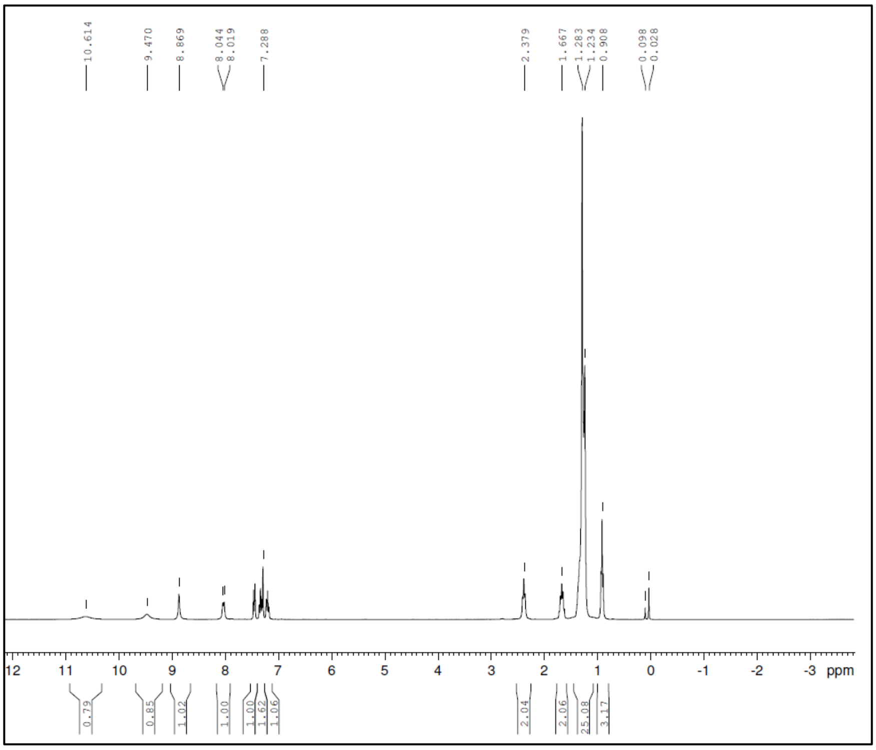

Figure S2. ^{1}H NMR spectrum of the product **2.**

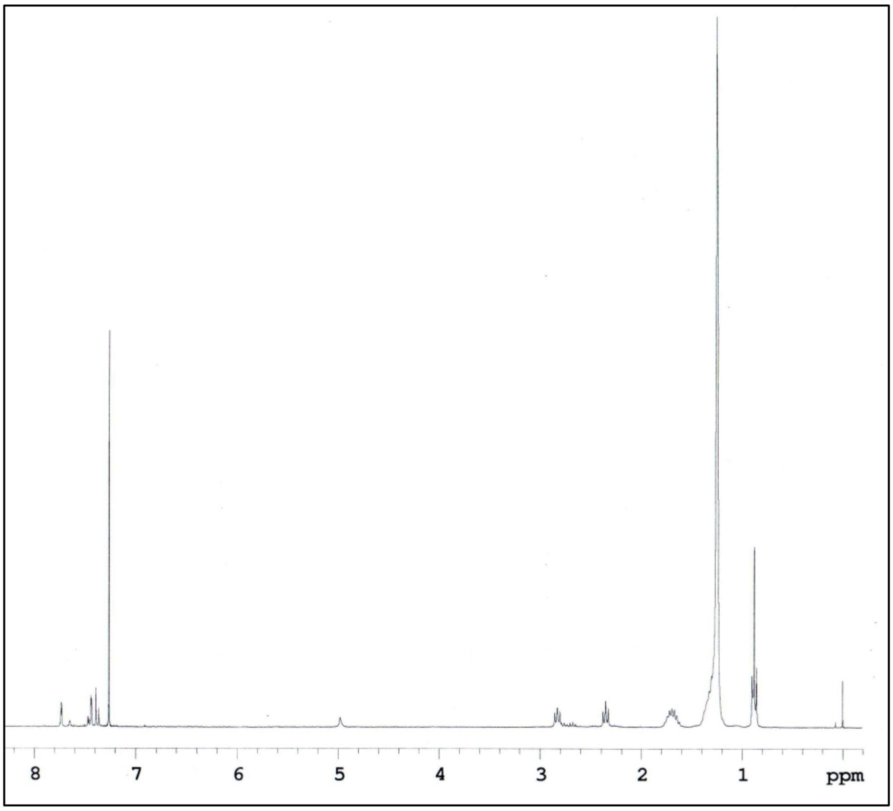

Figure S3. ^{1}H NMR spectrum of the product **3.**

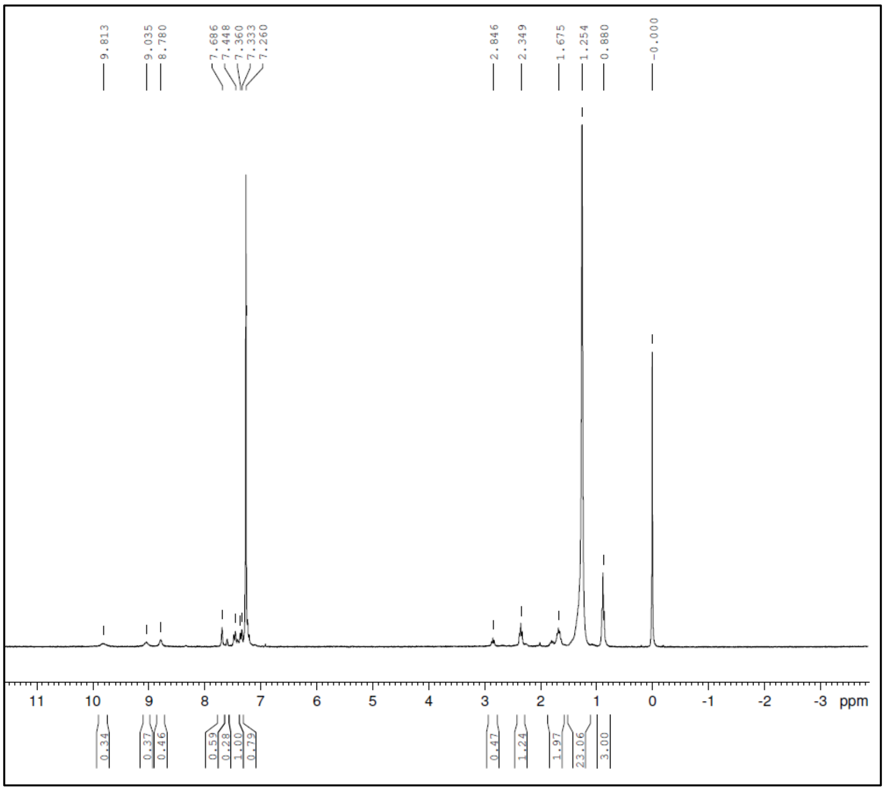

Figure S4. ^{1}H NMR spectrum of the product **4.**

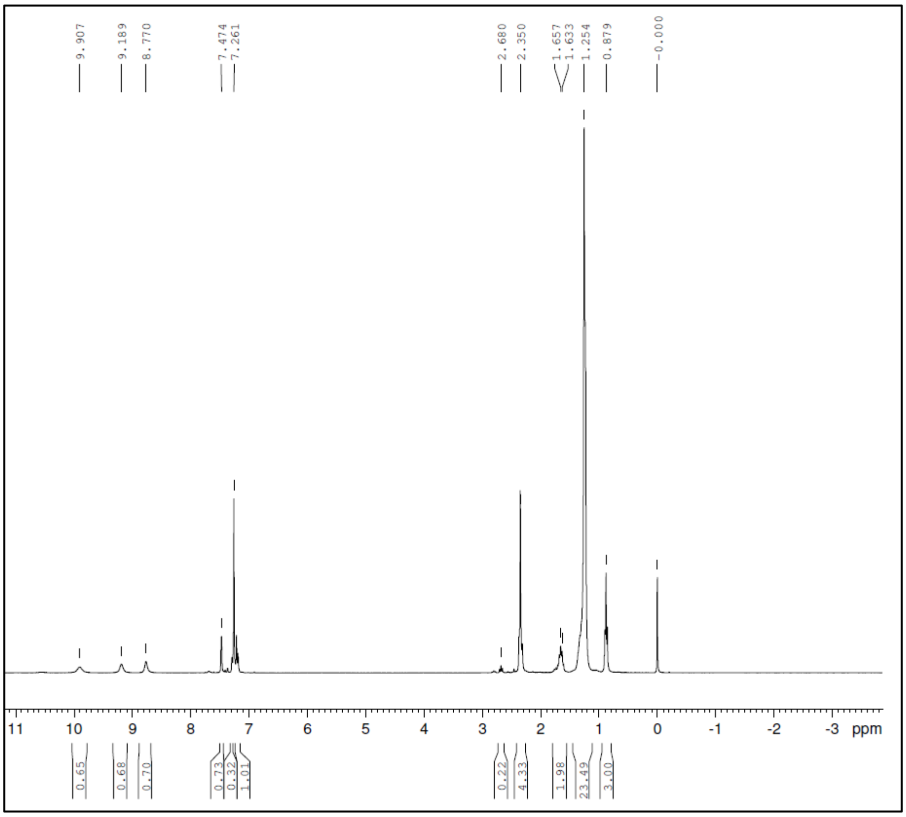

Figure S5. ^{1}H NMR spectrum of the product **5.**

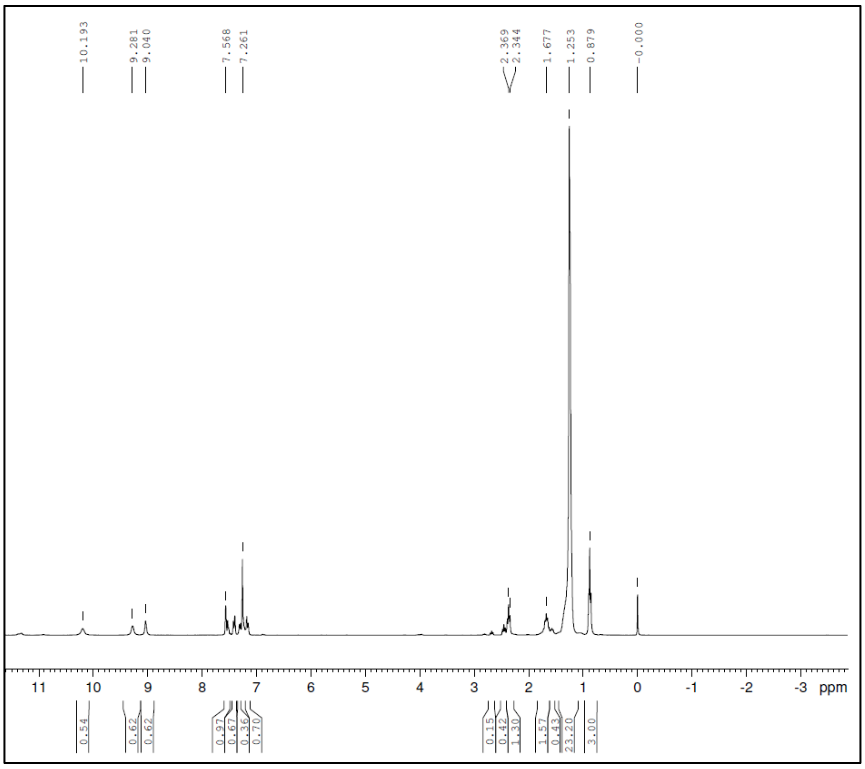

Figure S6. ^{1}H NMR spectrum of the product **6.**

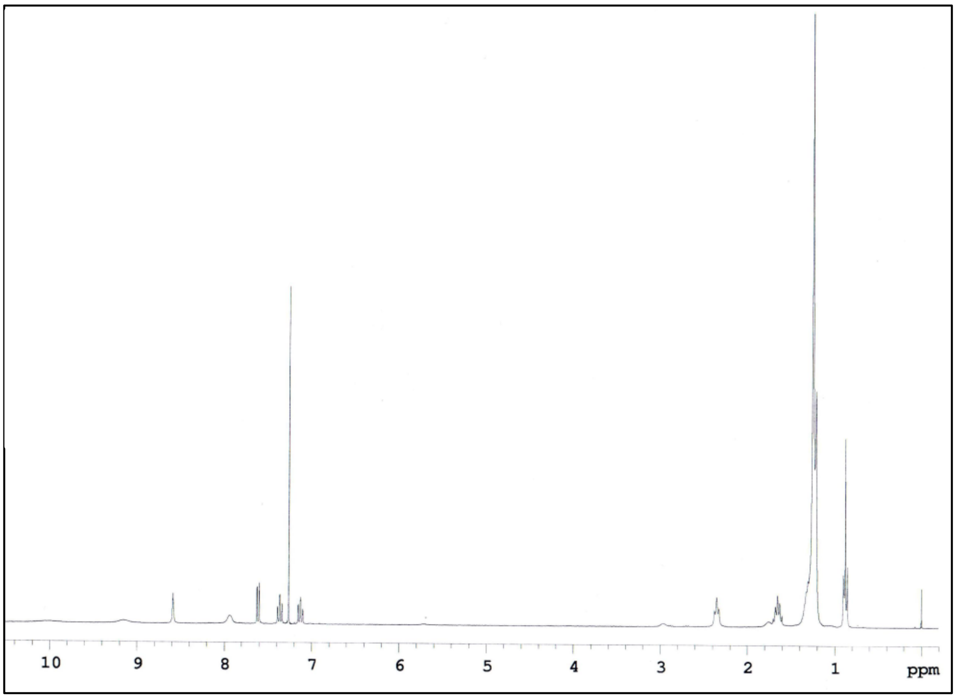

Figure S7. ^{1}H NMR spectrum of the product **7.**

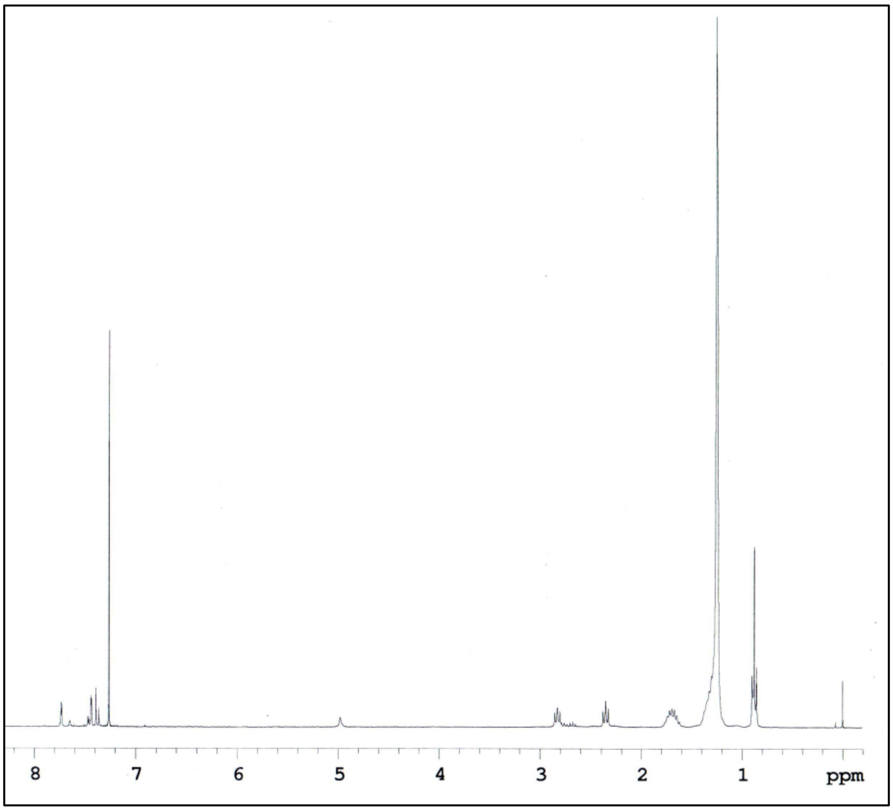

Figure S8. ^{1}H NMR spectrum of the product **8.**

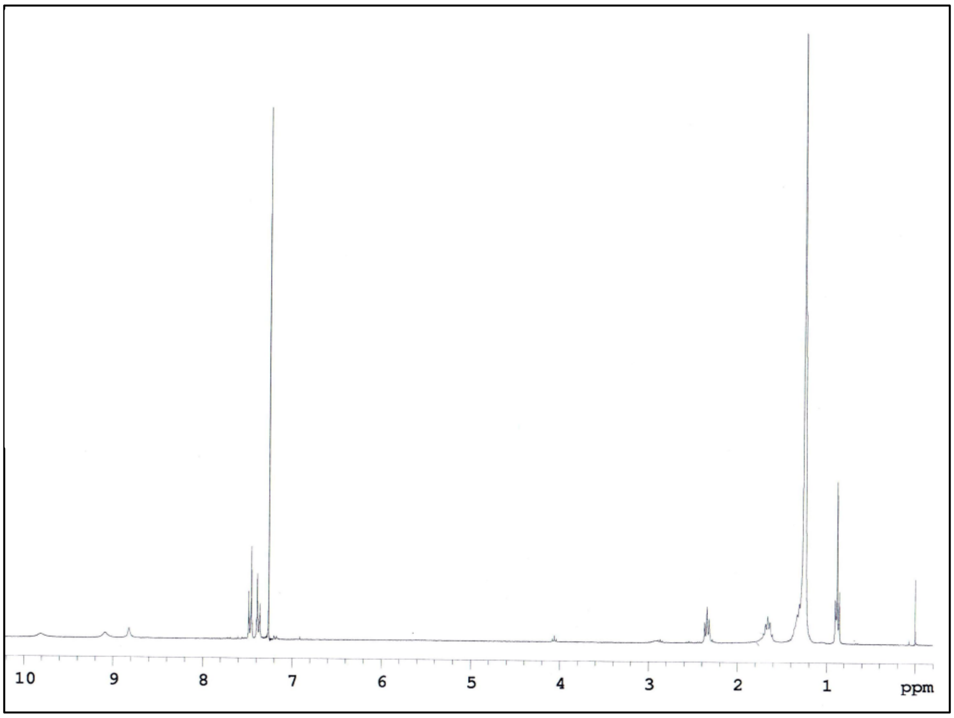

Figure S9. ^{1}H NMR spectrum of the product **9.**

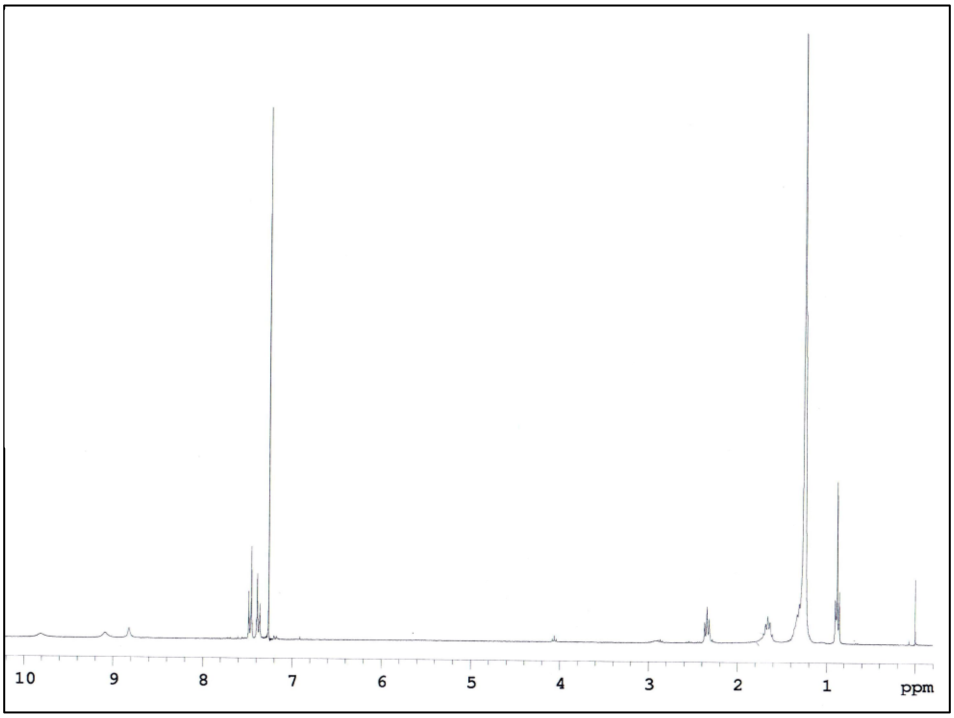

Figure S10. ^{1}H NMR spectrum of the product **10.**

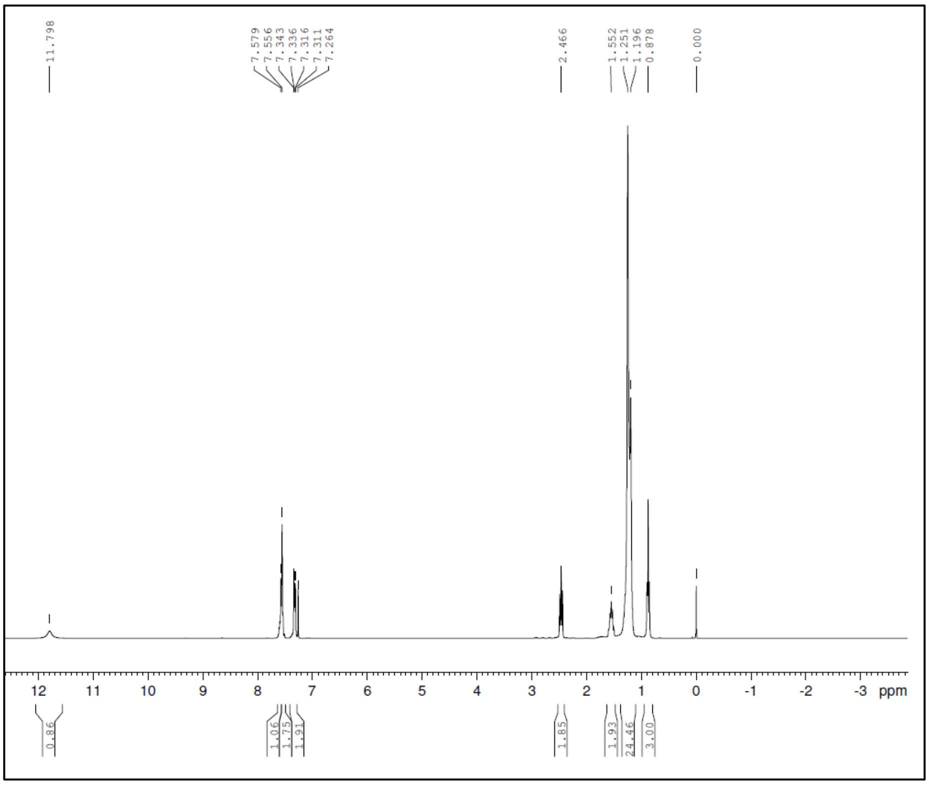

Figure S11. ¹H NMR spectrum of the product **1a.**

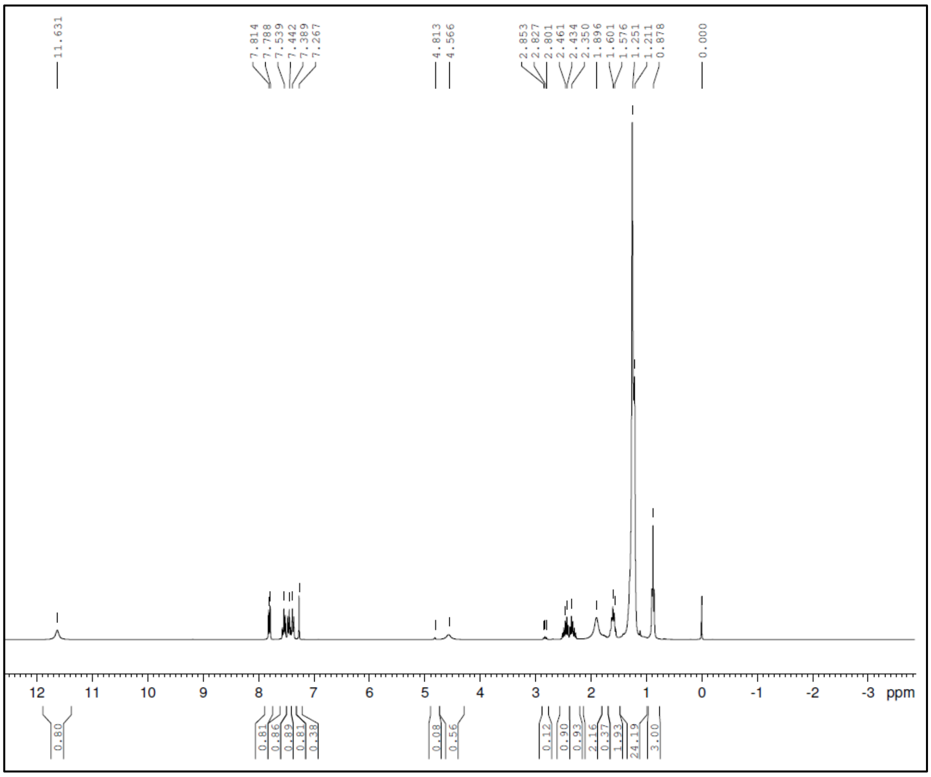

Figure S12. ¹H NMR spectrum of the product **2a.**

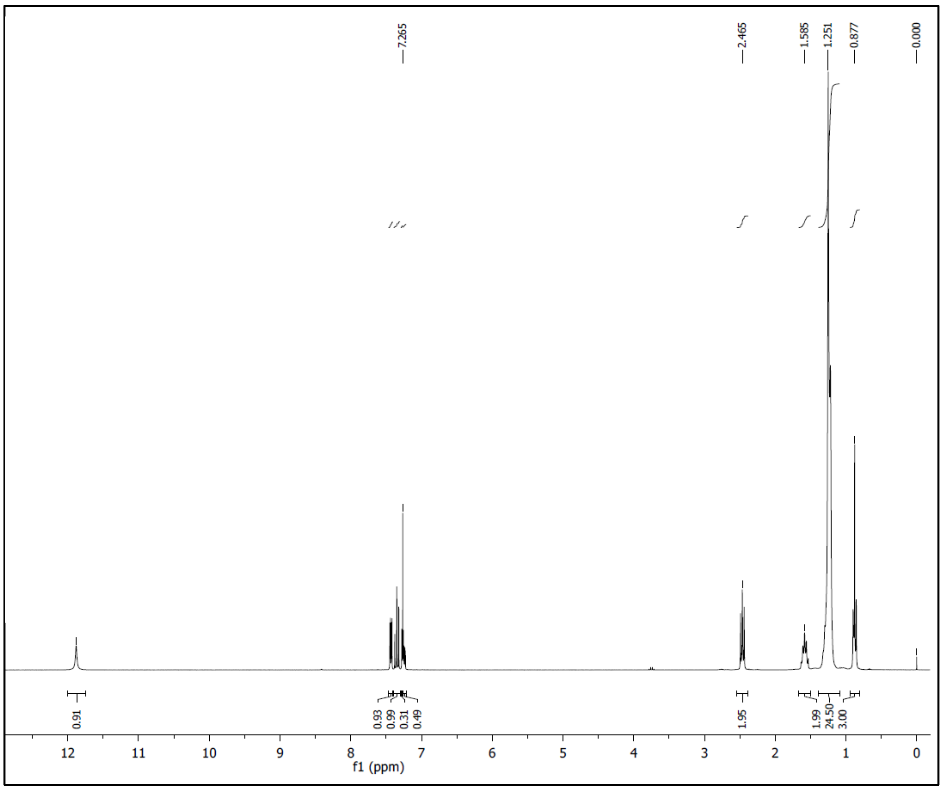

Figure S13. ^{1}H NMR spectrum of the product **3a.**

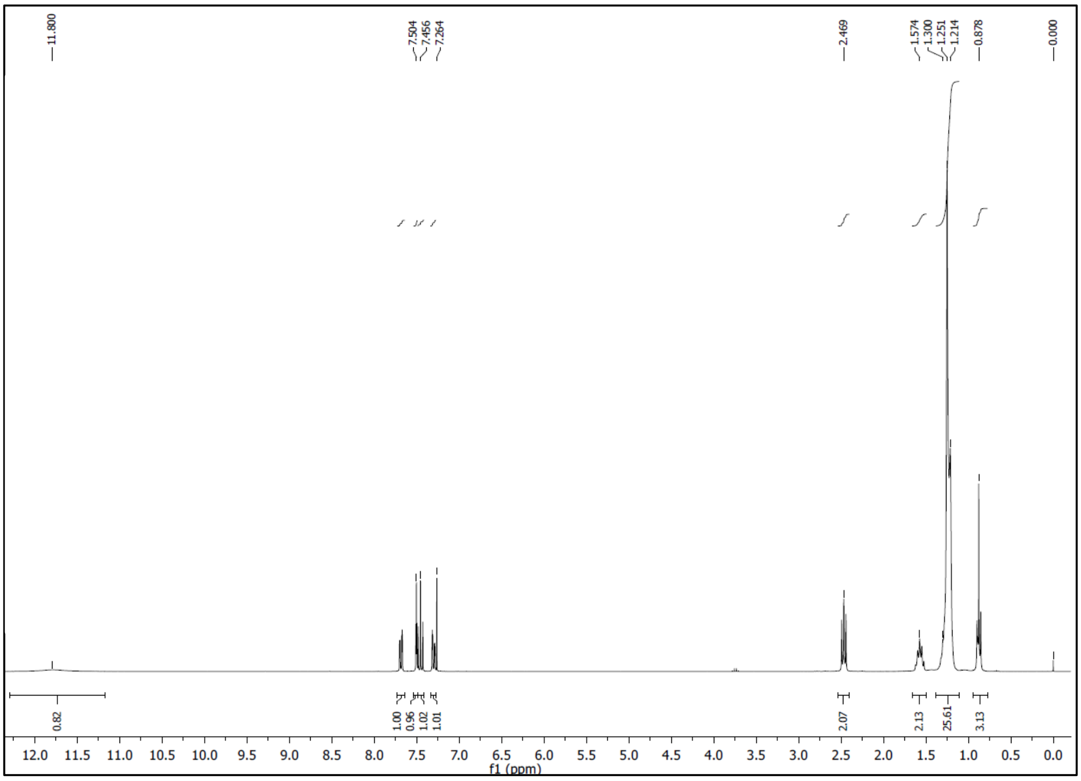

Figure S14. ^{1}H NMR spectrum of the product **4a.**

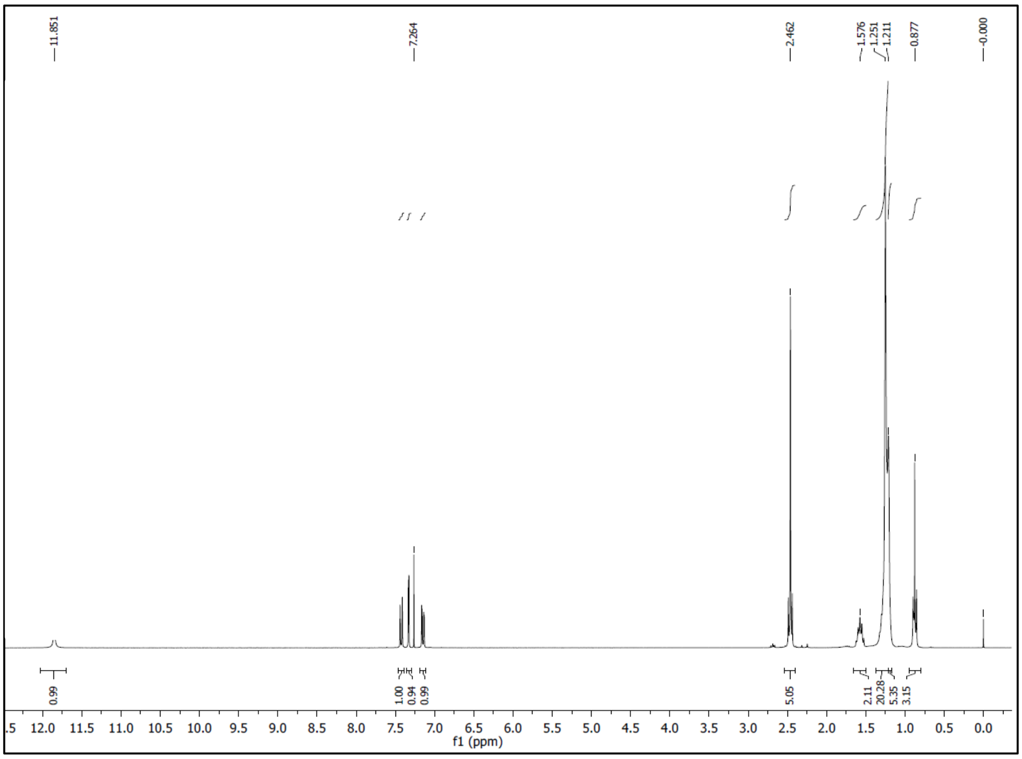

Figure S15. ^{1}H NMR spectrum of the product **5a.**

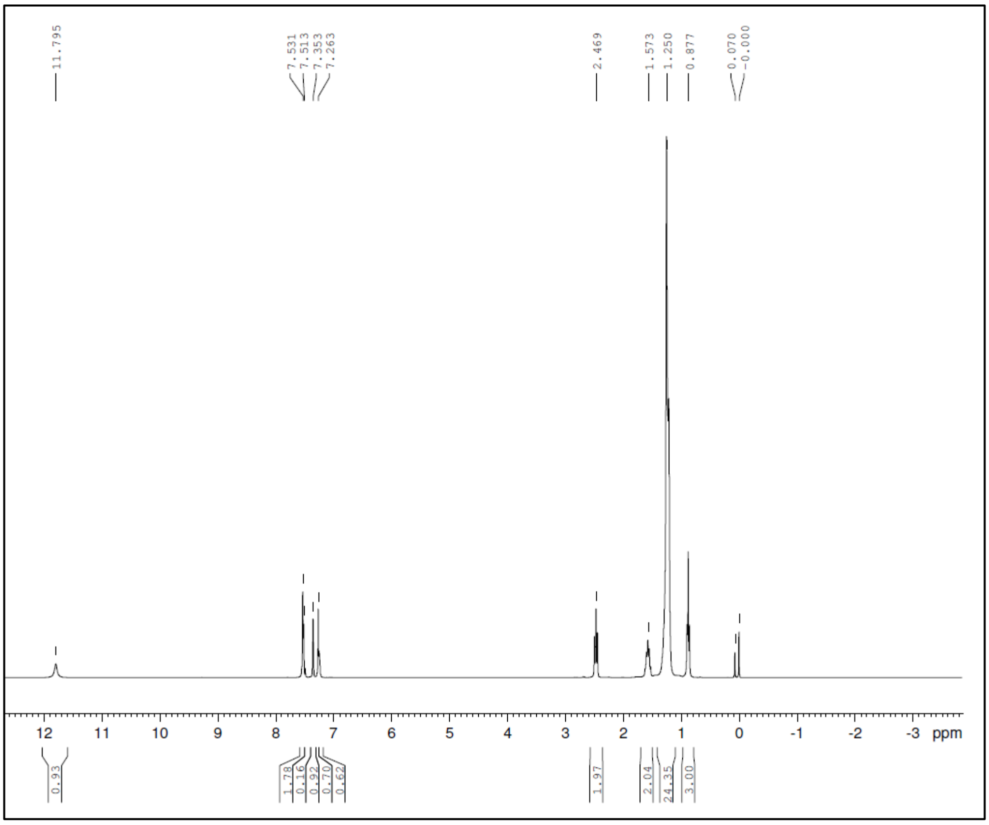

Figure S16. ^{1}H NMR spectrum of the product **6a.**

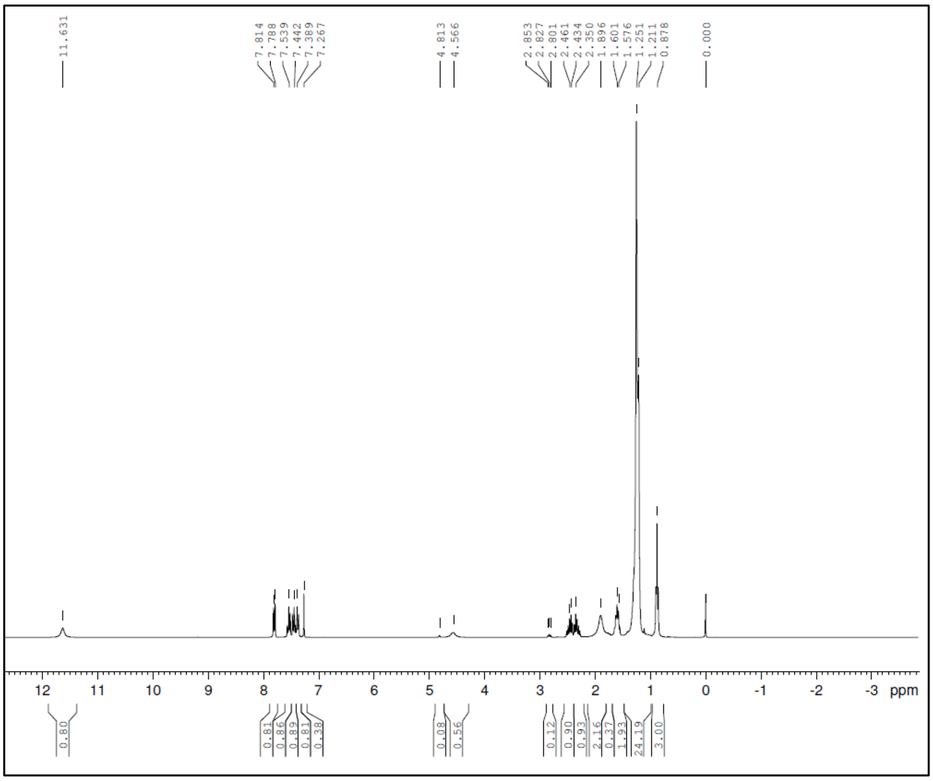

Figure S17. ^{1}H NMR spectrum of the product **7a.**

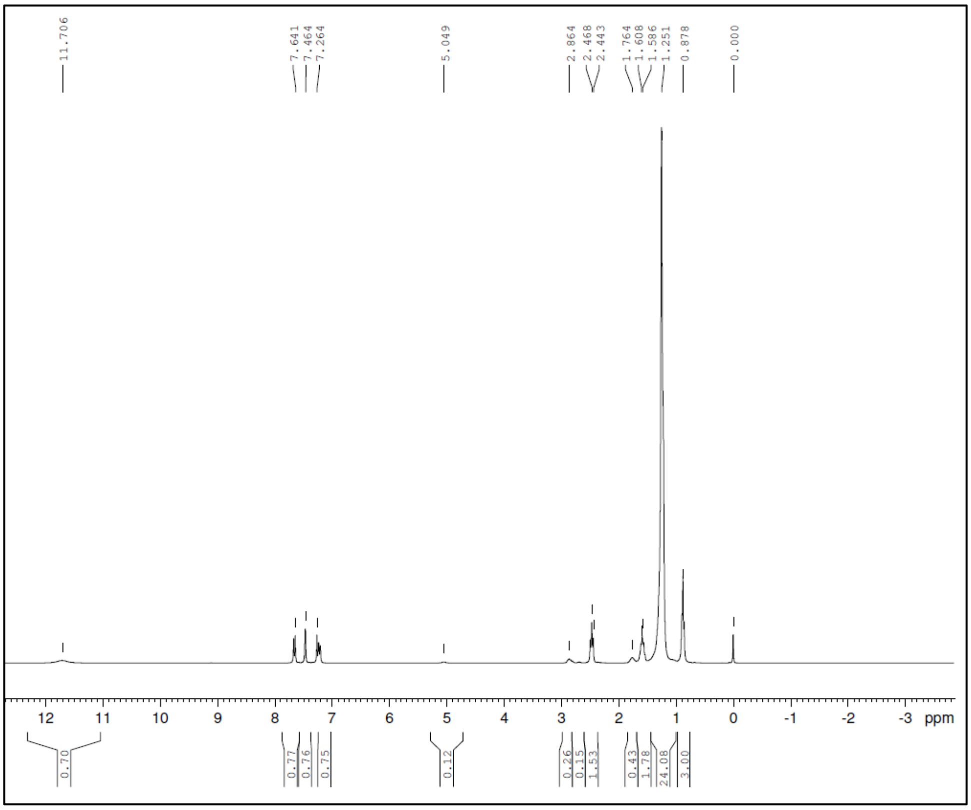

Figure S18. ^{1}H NMR spectrum of the product **8a.**

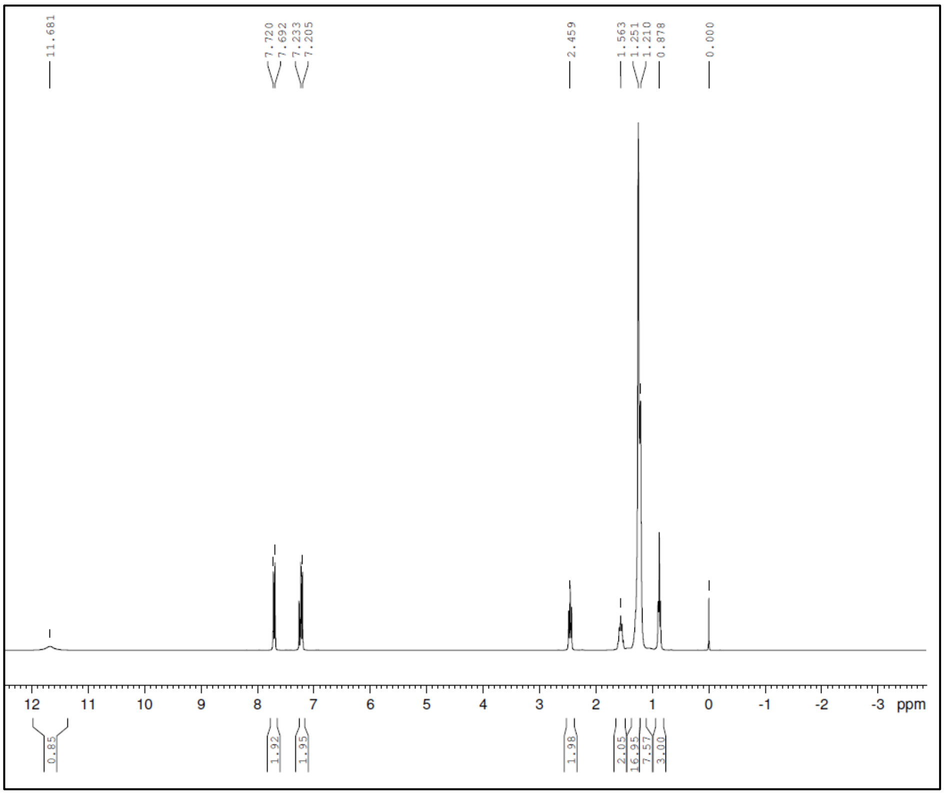

Figure S19. ^{1}H NMR spectrum of the product **9a.**

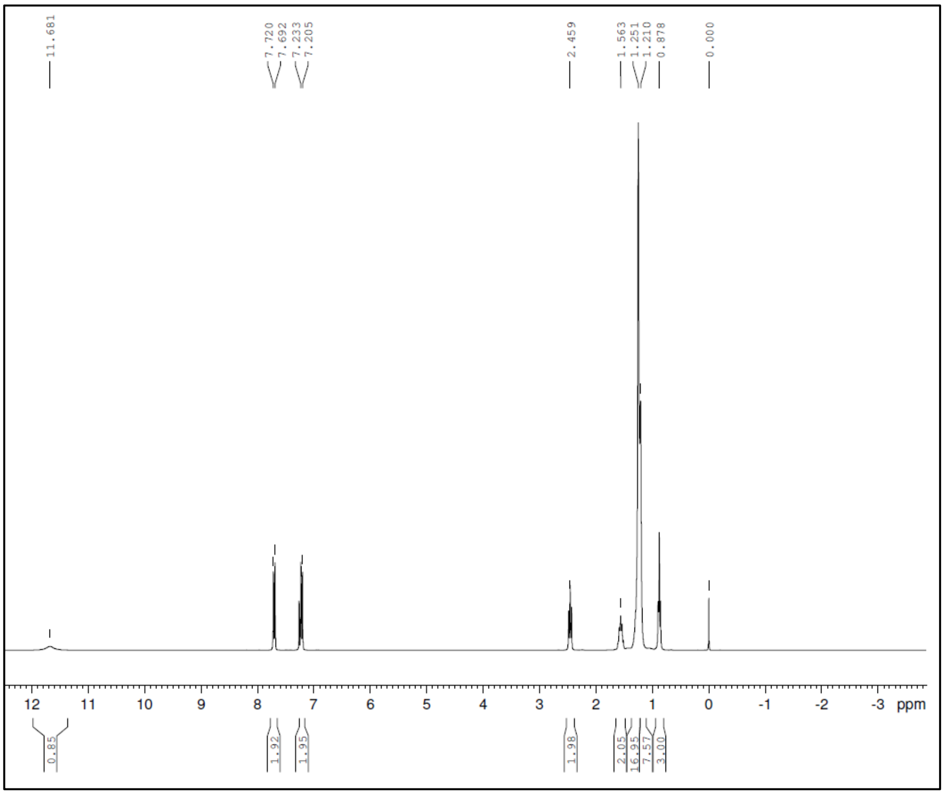

Figure S20. ^{1}H NMR spectrum of the product **10a.**

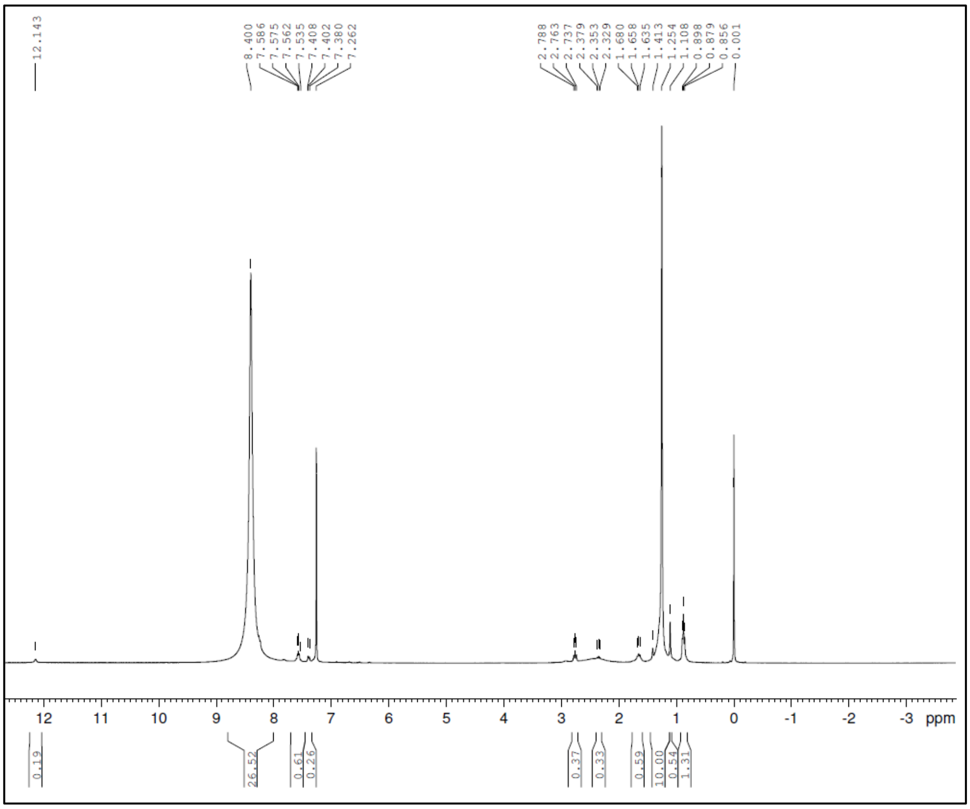

Figure S21. ^{1}H NMR spectrum of the product **1b.**

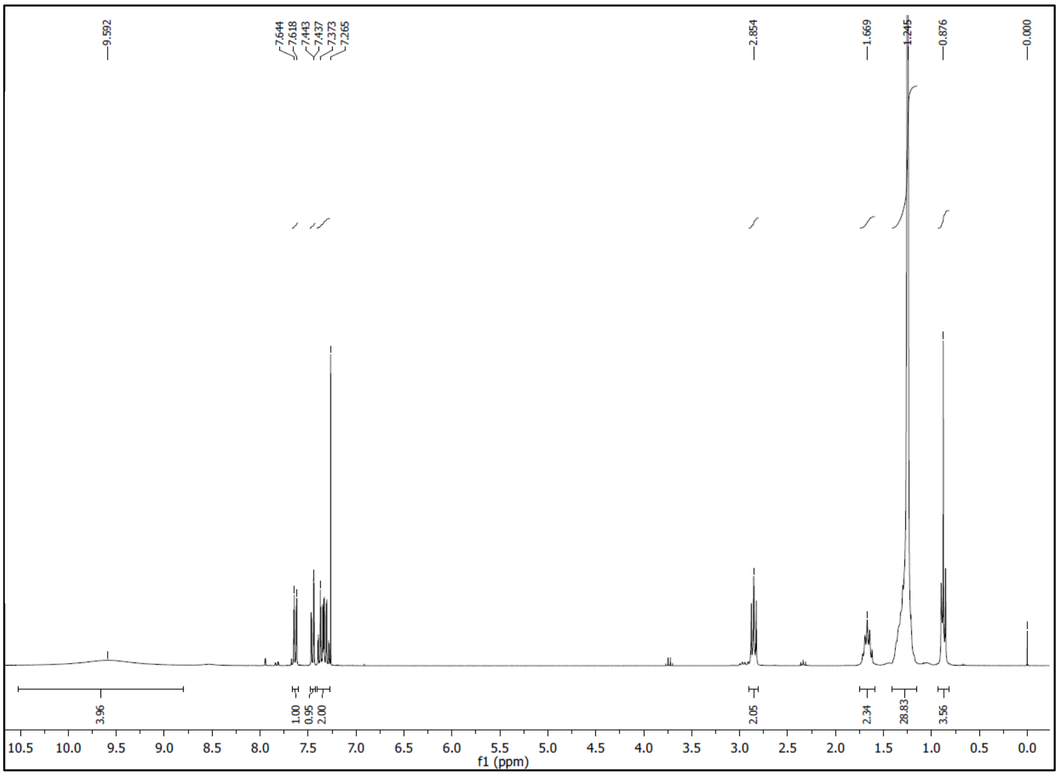

Figure S22. ^{1}H NMR spectrum of the product **2b.**

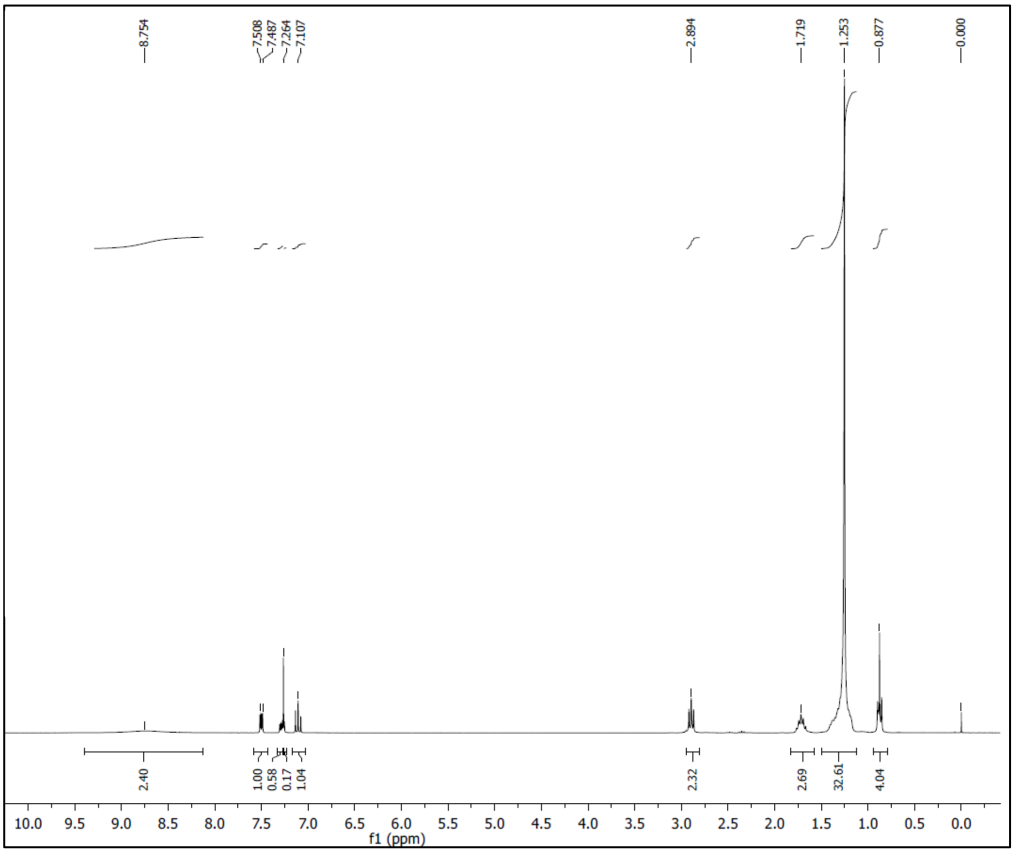

Figure S23. ^{1}H NMR spectrum of the product **3b.**

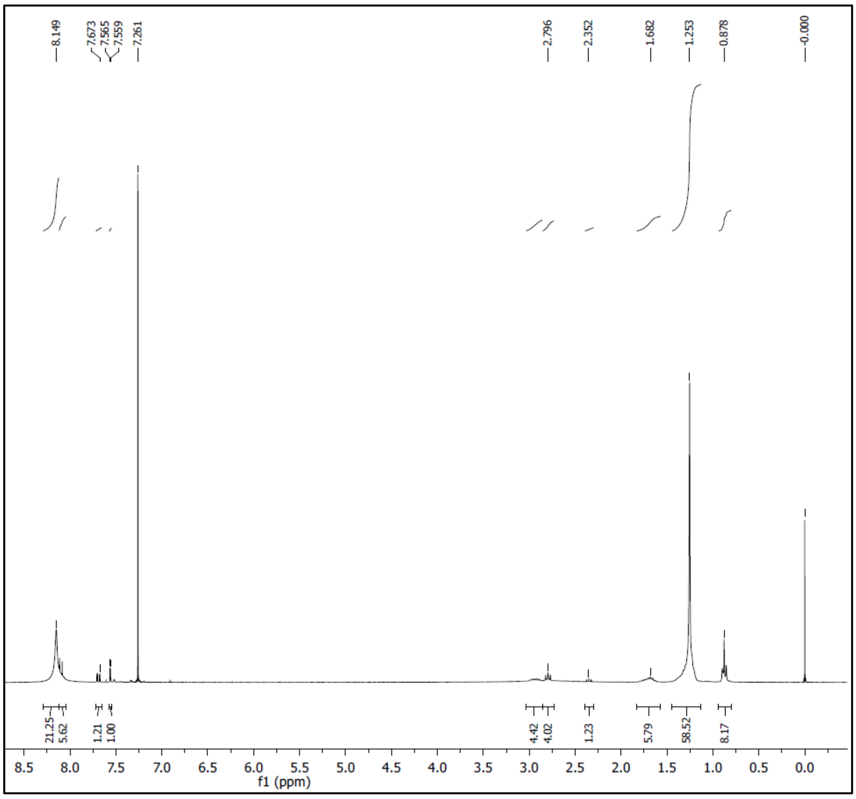

Figure S24. ^{1}H NMR spectrum of the product **4b.**

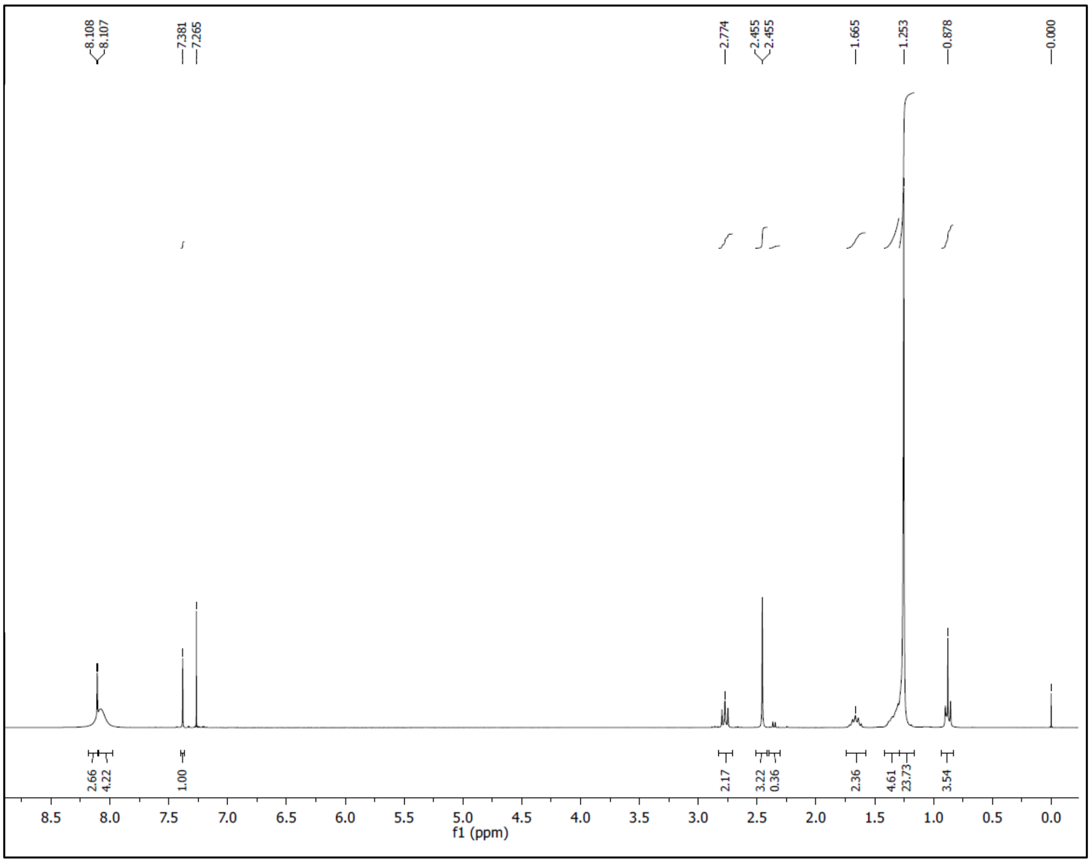

Figure S25. ¹H NMR spectrum of the product **5b.**

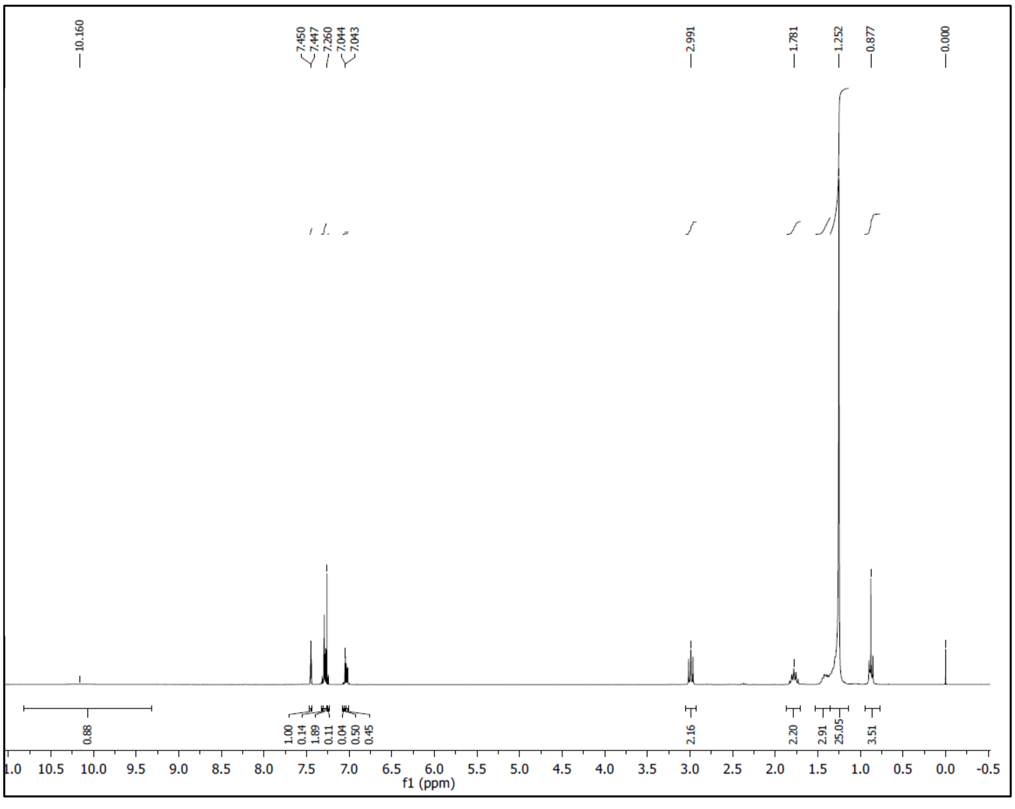

Figure S26. ¹H NMR spectrum of the product **6b.**

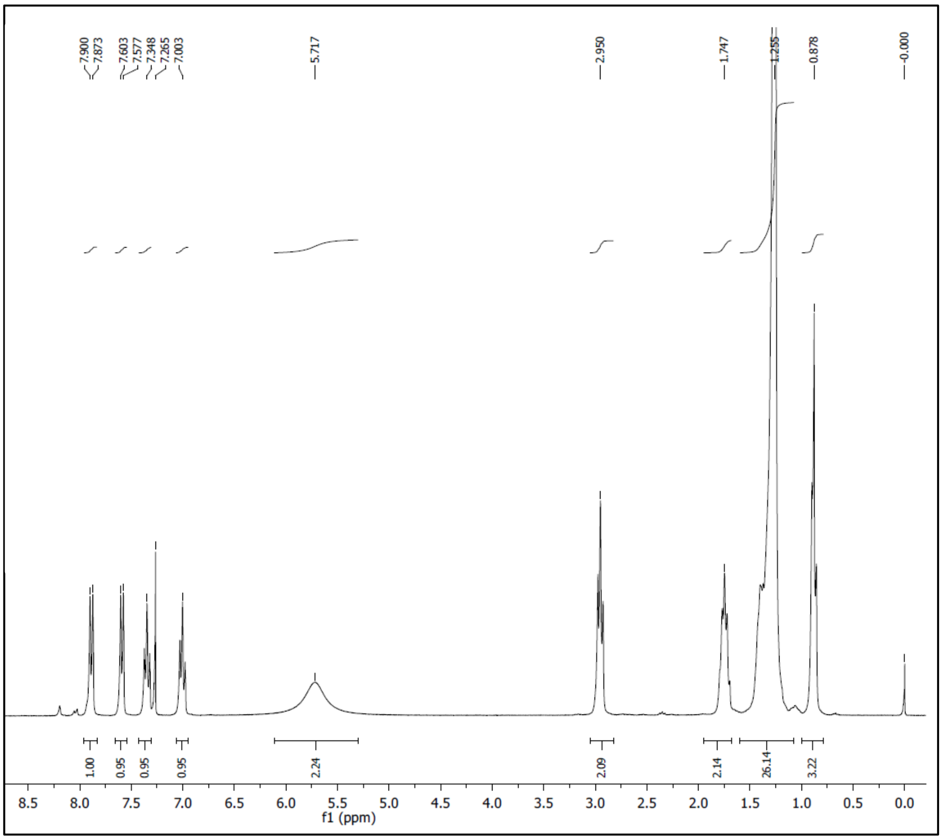

Figure S27. 1H NMR spectrum of the product **7b.**

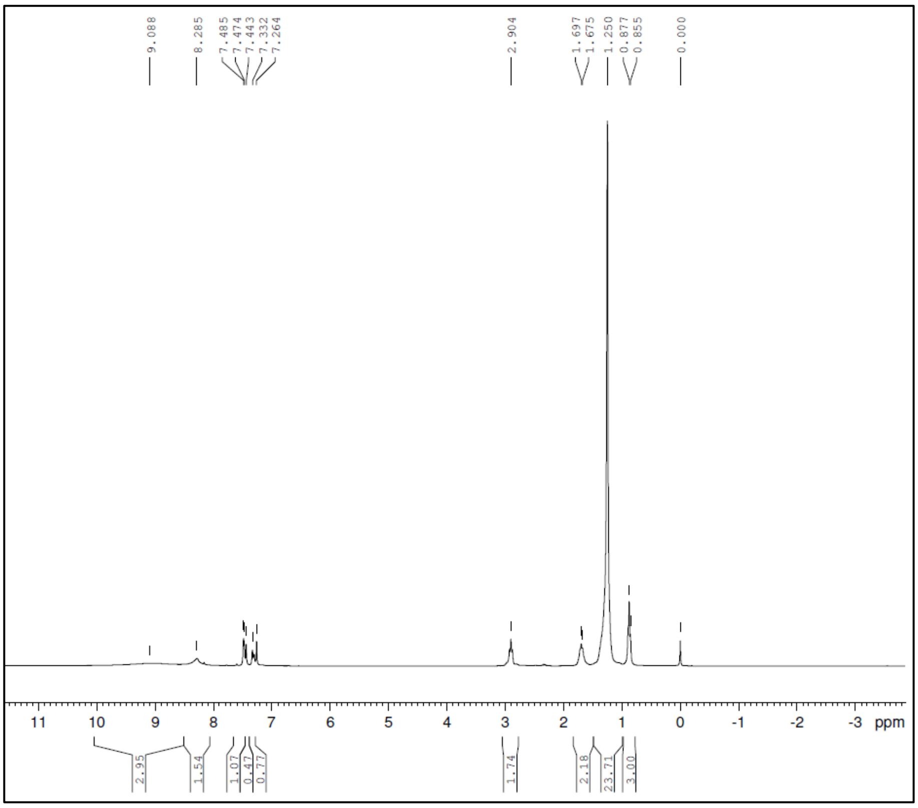

Figure S28. 1H NMR spectrum of the product **8b.**

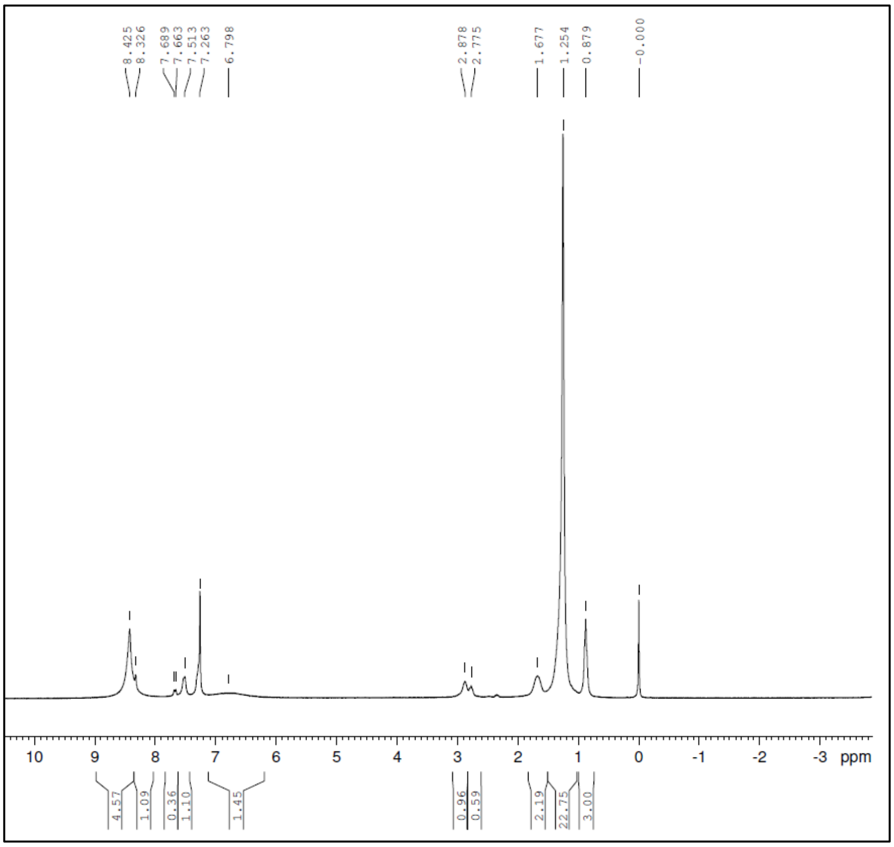

Figure S29. ^{1}H NMR spectrum of the product **9b.**

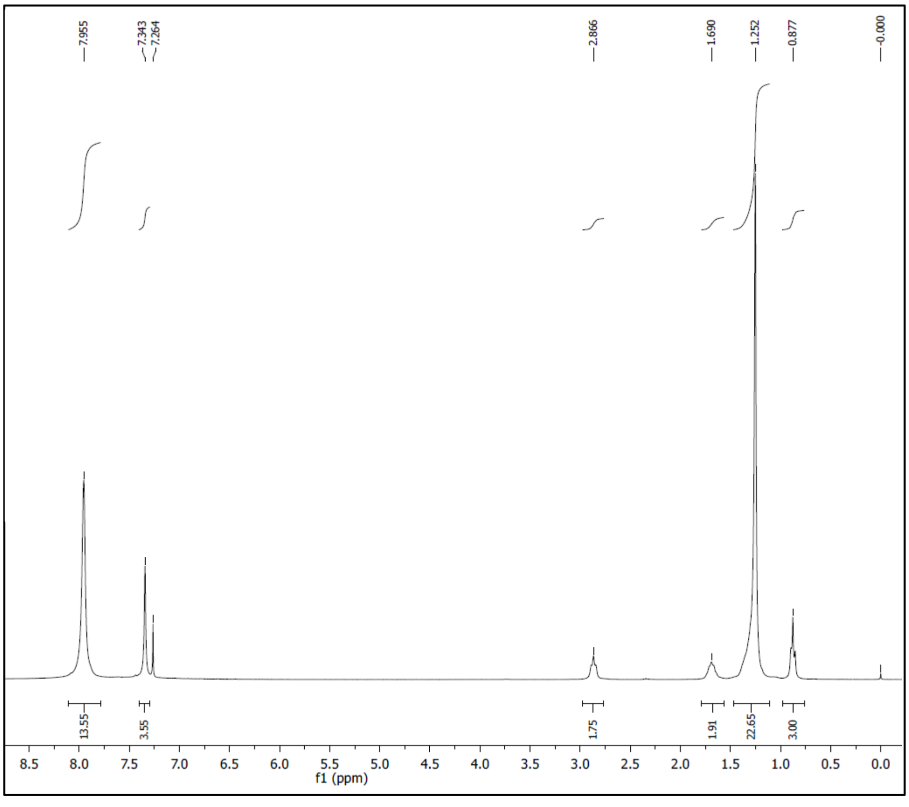

Figure S30. ^{1}H NMR spectrum of the product **10b.**

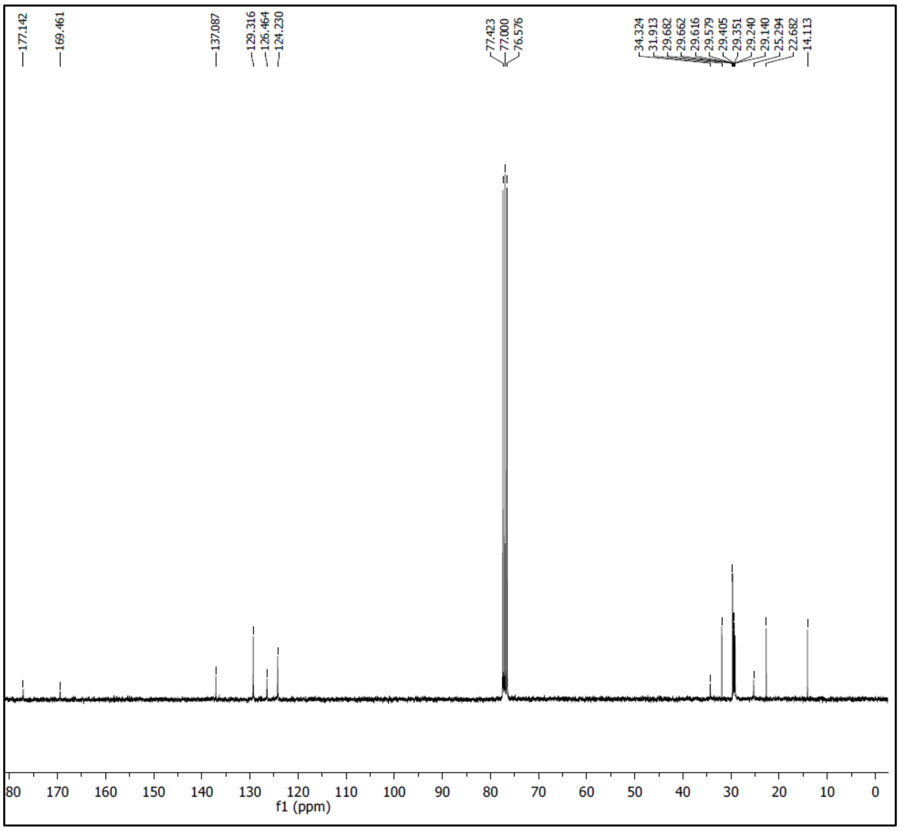

Figure S31. ^{13}C NMR spectrum of the product **1.**

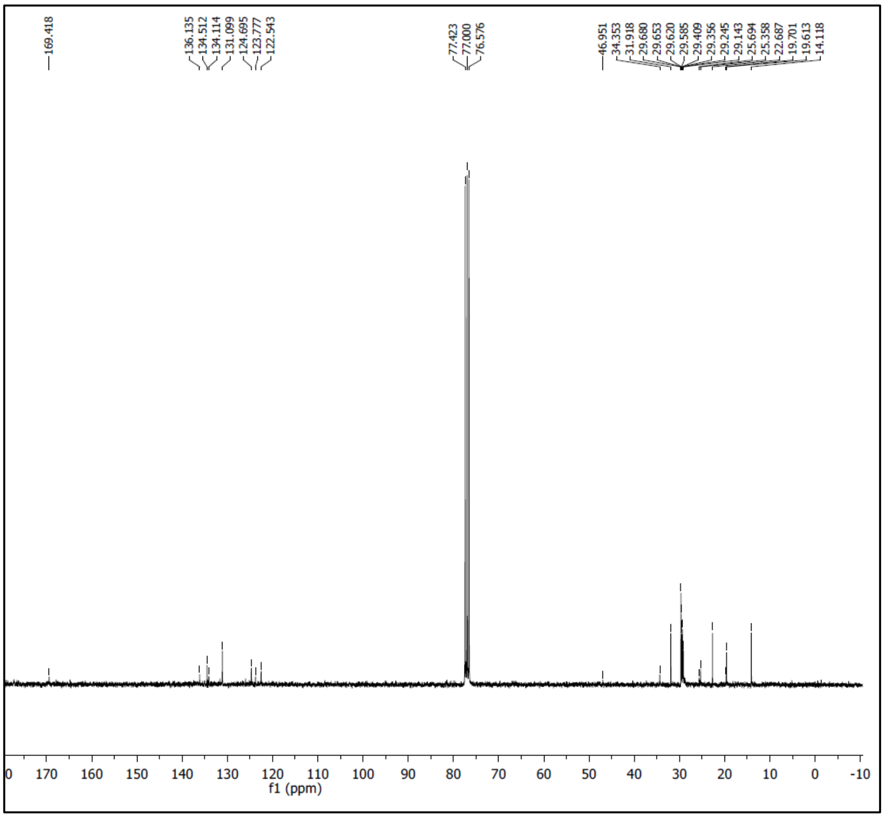

Figure S32. ^{13}C NMR spectrum of the product **5.**

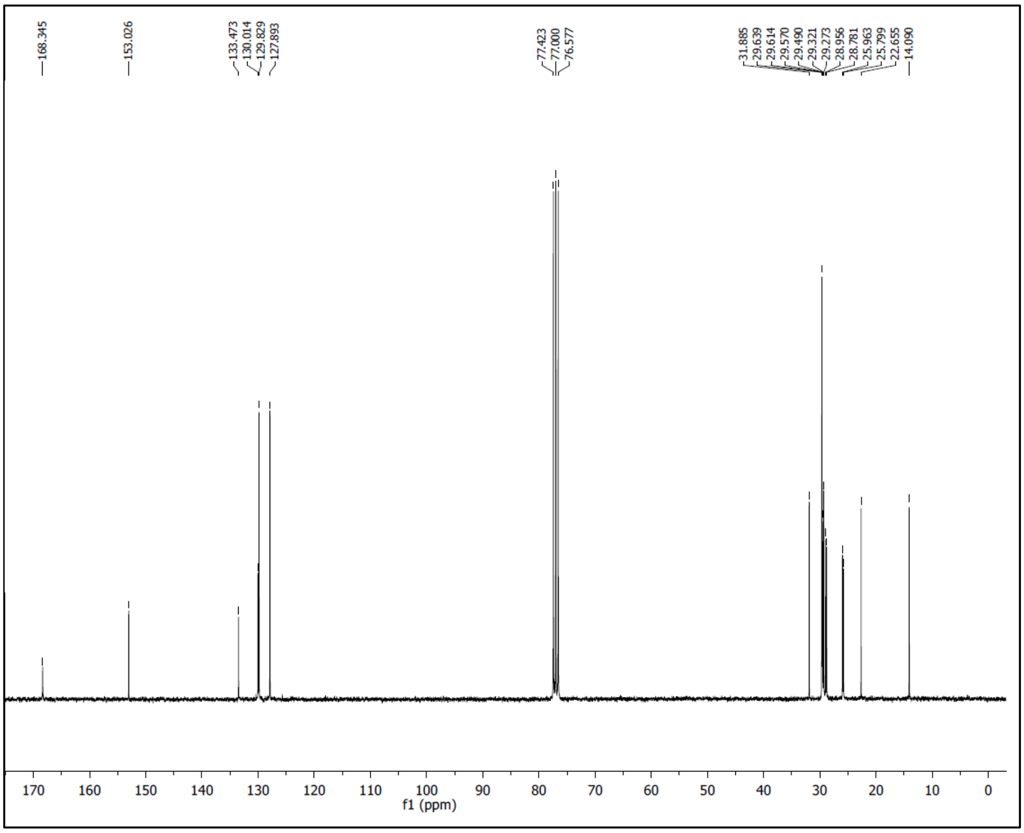

Figure S33. ^{13}C NMR spectrum of the product **1a.**

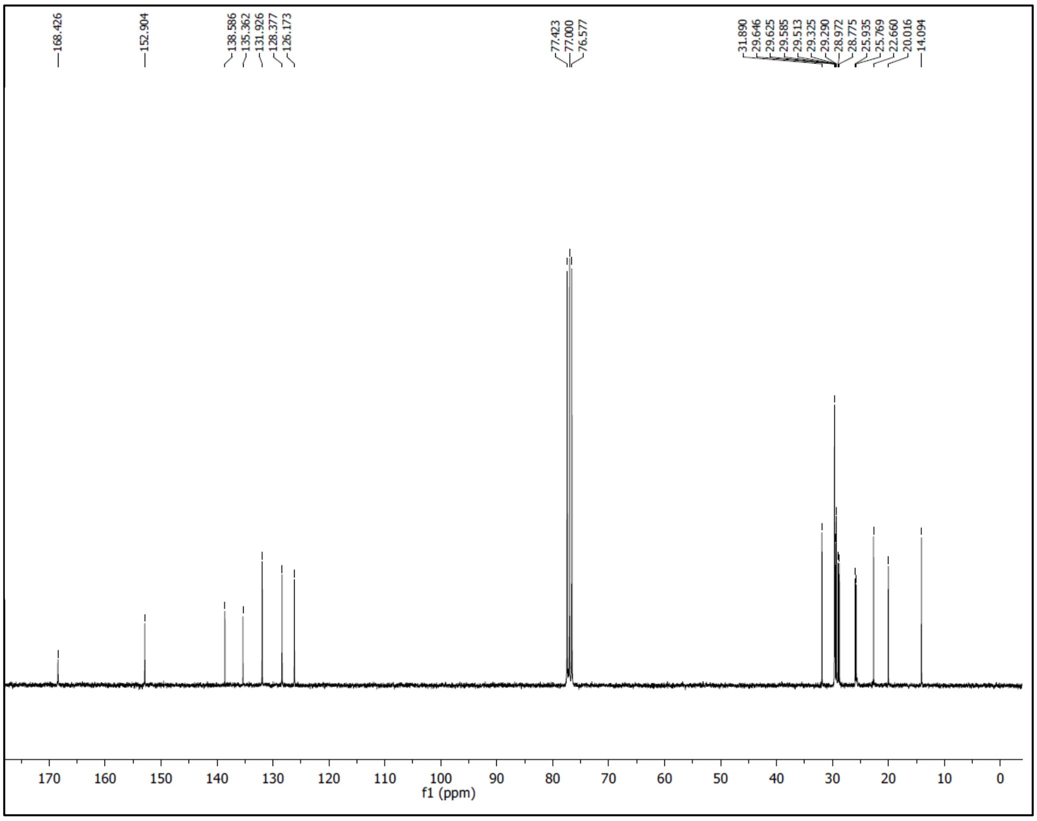

Figure S34. ^{13}C NMR spectrum of the product **5a.**

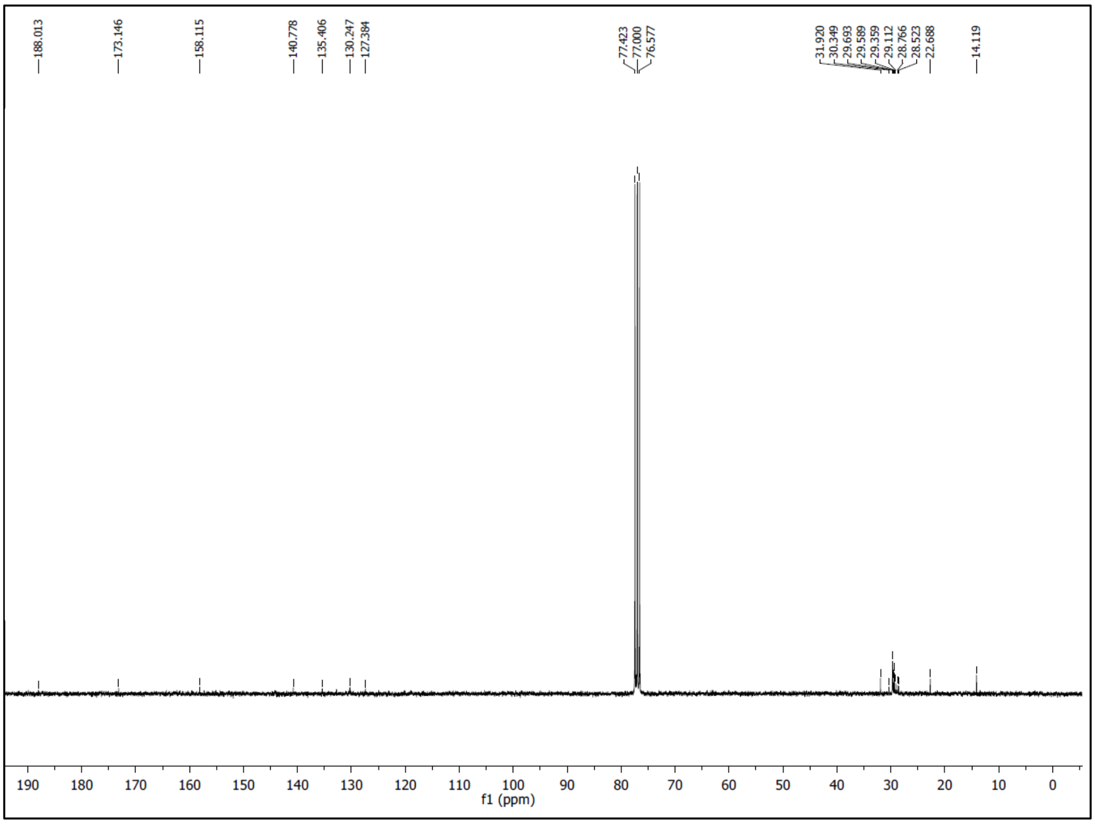

Figure S35. ^{13}C NMR spectrum of the product **1b.**

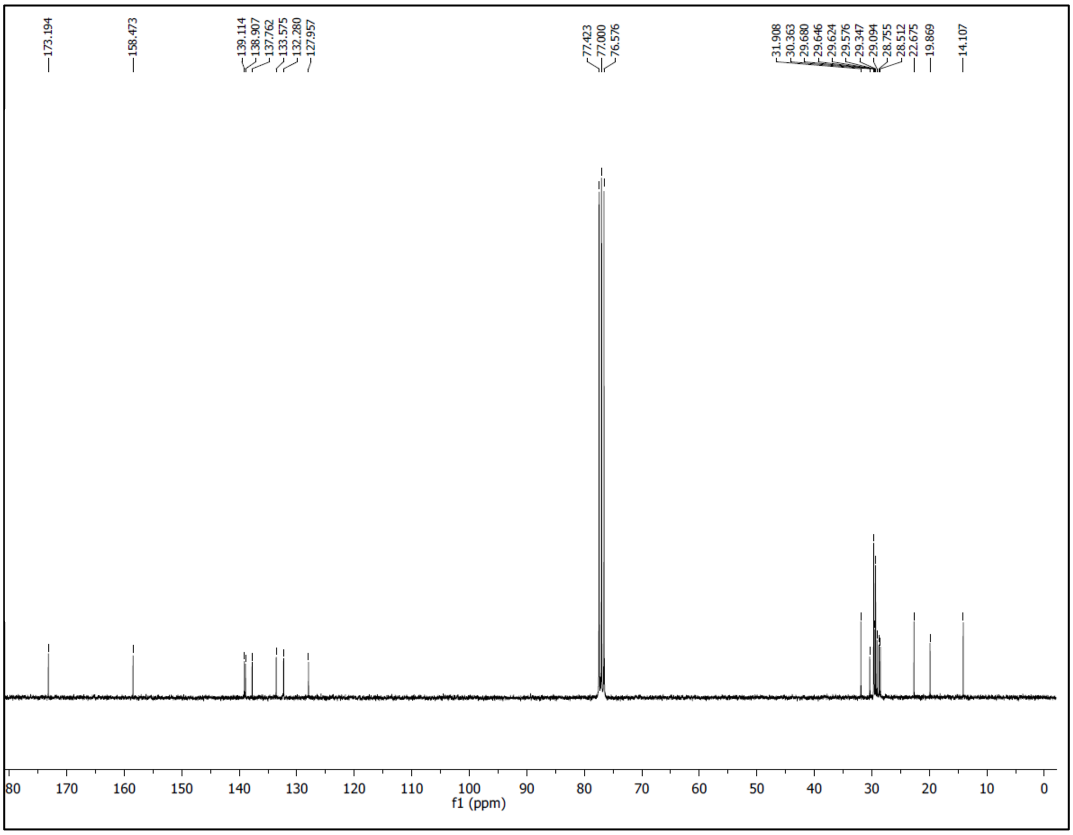

Figure S36. ^{13}C NMR spectrum of the product **5b.**
